# Investigation of *alpl* expression and Tnap-activity in zebrafish implies conserved functions during skeletal and neuronal development

**DOI:** 10.1038/s41598-020-70152-5

**Published:** 2020-08-07

**Authors:** Barbara Ohlebusch, Angela Borst, Tina Frankenbach, Eva Klopocki, Franz Jakob, Daniel Liedtke, Stephanie Graser

**Affiliations:** 1 Institute for Human Genetics, Biocenter, Julius-Maximilians-University, Würzburg, Germany; 2 Developmental Biology Unit, Institute of Zoology, University of Cologne, Cologne, Germany; 3 Bernhard-Heine-Center for Locomotion Research, Julius-Maximilians-University, Würzburg, Germany

**Keywords:** Bone development, Neurogenesis, Development

## Abstract

Hypophosphatasia (HPP) is a rare genetic disease with diverse symptoms and a heterogeneous severity of onset with underlying mutations in the *ALPL* gene encoding the ectoenzyme Tissue-nonspecific alkaline phosphatase (TNAP). Considering the establishment of zebrafish (*Danio rerio*) as a new model organism for HPP, the aim of the study was the spatial and temporal analysis of *alpl* expression in embryos and adult brains. Additionally, we determined functional consequences of Tnap inhibition on neural and skeletal development in zebrafish. We show that expression of *alpl* is present during embryonic stages and in adult neuronal tissues. Analyses of enzyme function reveal zones of pronounced Tnap-activity within the telencephalon and the mesencephalon. Treatment of zebrafish embryos with chemical Tnap inhibitors followed by axonal and cartilage/mineralized tissue staining imply functional consequences of Tnap deficiency on neuronal and skeletal development. Based on the results from neuronal and skeletal tissue analyses, which demonstrate an evolutionary conserved role of this enzyme, we consider zebrafish as a promising species for modeling HPP in order to discover new potential therapy strategies in the long-term.

## Introduction

Hypophosphatasia (HPP) is a rare hereditary disease with a diversity of systemic manifestations including a predominant skeletal phenotype. The genetic cause of HPP are mutations in the *ALPL* gene, which encodes the ectoenzyme tissue-nonspecific alkaline phosphatase (TNAP). The disease is classified due to the age of diagnosis and the severity of onset^1^. Clinical HPP manifestations show a broad spectrum of severity depending on the degree of *ALPL* deficiency, ranging from stillbirth or perinatal lethality to severe or moderate manifestations in childhood, adolescence and adulthood to the odonto-form of HPP that is restricted to teeth^1^. Apart from frequently occurring mineralization defects of bones and teeth, premature loss of deciduous teeth and craniosynostosis presuppose additional therapy for affected patients^1^. Moreover, neurological symptoms such as epileptic seizures, depression, sleep- or anxiety-disorders further hamper HPP-patients’ quality of life^2^. The prevalence for severe HPP forms has been estimated 1:300.000 and 1:6,370 for moderate forms in the European population^3^. To date 409 mutations (including 71.4% missense mutations) localized in the human *ALPL* gene have been listed in the database that is provided by E. Mornet (https://www.sesep.uvsq.fr/03_hypo_mutations.php; 7th of May 2020). The mode of inheritance for HPP is either autosomal dominant or recessive, whereas the latter is common for severe cases and both traits have been described for mild onsets of the disease^3^. Additionally, dominant negative mutations are frequently reported in the context of HPP and its underlying *ALPL* mutations^3^. Genotype–phenotype correlations have been published, however cases have been described where same genotype leads to different severity of symptoms^4,5^.

Different tissues express different alkaline phosphatase (AP) isoforms and HPP patients’ phenotypes can greatly differ in the responsiveness between tissues^1^. Remarkably, comparative studies have already shown that the predominant TNAP transcript variant in bones is the same as in brain tissues^6^, which implies a common mode of TNAP function in both tissues. TNAP’s most important biochemical function in bones and teeth is providing the basis for mineralization processes. The enzyme enables hydroxyapatite crystallization in bone via catalyzing the dephosphorylation of mineralization inhibitors such as inorganic pyrophosphate (PP_i_) and phosphorylated osteopontin^7^. In the nervous system, two predominant mechanisms, the availability of vitamin B6 and alterations in purinergic signaling^8,9^, significantly influence the outcome of the disease. In case of decreased TNAP-activity, pyridoxal-5-phosphate (PLP), which is the transportable form of vitamin B6, accumulates within the serum and cannot be redistributed into the brain without dephosphorylation via TNAP. PLP is an essential enzymatic co-factor within central neurotransmitter synthesis pathways in the brain and the lack of PLP results in neurological impairment due to limited biochemical conversion^8^. Additionally, TNAP enzyme supports the development and maintenance of synapse functionality and is involved in the outgrowth and myelination of neurites^10–12^. Localization of TNAP within primates’ brains has been described as distinct patterns within layer 4 and 5 of the cortex^13,14^ and has been detected in the retina across a number of different vertebrate species^15^. In mice, TNAP localization was detected predominantly in endothelial cells, primordial germ cells, pioneer growth cones, and neural precursors^16^. *Akp2* (the murine version of the human *ALPL* gene) knockout mice display reduced serum levels of alkaline phosphatase, elevated substrate levels, impaired bone mineralization, and frequently die from epileptic seizures^17,18^. Due to the early lethal phenotype in mice and the lack of supplementary in vivo studies within other vertebrates, our knowledge about TNAP’s interconnected functions within bone and neuronal tissues is still scarce.

Due to a number of biological properties, the zebrafish (*Danio rerio*) has become an important vertebrate model organism for bone^19,20^ and brain research^21^. Along with other advantages, including low cost and easy housing of the animals, the possibility to investigate transgenic reporter lines by non-invasive in vivo monitoring and perform large scale screenings, zebrafish offer a wide repertoire of different approaches for investigation of bone and brain development^22–24^. Furthermore, basic molecular pathways for vertebrate development, like bone development, are evolutionarily conserved and share common properties with other species^25,26^. Consequently, a rising number of in vivo models for musculoskeletal diseases have been established in recent years^27–29^ and even the molecular background of complex diseases, such as craniosynostosis, has been faithfully reproduced in a zebrafish model^30–32^. Despite the analysis of mineralization and skeletal issues, feasible strategies for the investigation of complex neurological diseases, such as anxiety disorder and depression, have already been successfully modeled within the zebrafish^33–37^. Comparison of multiple neurological functions linked to the amygdala is one example, which has been attributed to being relevant for anxiety in a wide number of different vertebrate species^35^. Furthermore, novel genetic and visualization techniques primarily established in zebrafish enable detailed cellular resolution of gene expression during brain development and open up the opportunity to image neural activity in freely behaving larvae^38,39^.

Collectively, the conservation of essential signaling pathways and developmental processes, high evolutionary conservation between the TNAP/Tnap encoding genes^40,41^, and advanced imaging, emphasize zebrafish as a promising vertebrate disease model for HPP research by providing new insights into currently unsolved questions of HPP research and consequently paving the way for the development of new therapeutic approaches for this rare disease. Finally, the fact that the molecular basis of most neurological and a number of HPP symptoms in bones, such as the development of craniosynostosis, have not been elucidated in sufficient detail yet, makes future research indispensable.

## Materials and methods

### Zebrafish maintenance

Zebrafish (*Danio rerio*) were bred and maintained as previously described^42–44^ in the aquatic facilities of the Biocenter of the Julius-Maximilians-University Würzburg, Germany according to FELASA guidelines. In short, adult fish are kept at a mean temperature of 24–26 °C in 10 l glass and 2.5 l plastic tanks, while embryos younger than 120 h post-fertilization (hpf) are raised at a temperature of 28.5 °C in an incubator. A daily light cycle of 10 h dark/14 h light is maintained. Preconditioned reverse osmosis water with adjusted conductivity 800–1,100 µS/cm, pH7.0 and stable water hardness is used. A food combination of *Artemia nauplii* and GEMMA Micro Food (age dependent sizes; Skretting, USA) is standard.

All experimental procedures were performed according to the guidelines of the German animal welfare law and approved by the local government (Government of Lower Franconia; Tierschutzgesetz §11, Abs. 1, Nr. 1, husbandry permit number 568/300-1870/13). Used zebrafish strains: *AB/TU* (ZDB-GENO-010924-10), *AB/AB* (ZDB-GENO-960809-7) and *nac/tra* (*mitfa*^*w2/w2*^; *mpv17*^*b18/b18*^; ZFIN ID: ZDB-GENO-121010-3). Embryos were staged by morphological characteristics according to literature^42^. The denomination “hpf” indicates hours post fertilization at 28.5 °C.

### RNA isolation, reverse transcription and quantitative real-time PCR (qPCR)

For qPCR experiments, RNA was extracted from adult zebrafish organs (age 3–6 month; *AB/AB* strain). For cDNA synthesis, 1 µg RNA was transcribed into cDNA and further analyzed in a ViiA7 Real-Time PCR System (Thermo Fisher Scientific, Waltham, USA). Each group and primer sample was analyzed via qPCR in triplicates on a single plate utilizing HOT FIREPol Eva Green Mix Plus (Solis BioDyne). Data analysis was performed via QuantStudio Real-Time PCS Software v1.1 by ΔΔCt method. Muscle tissue served as reference sample for relative comparison. Amplification of *gapdh* and *eef1a1l1* was used as endogenous cDNA controls (housekeeping genes). Used primer pairs and targeted regions are noted in Table S1. Additional details on experimental set-up is given in the supplementary methods.

### Determination of alkaline phosphate activity in protein lysates

Organs of adult fish were extracted and instantly frozen in liquid nitrogen (age 3–6 month; *AB/AB* strain). Samples were stored at − 80 °C until experimental analyses. For protein extraction, samples were disrupted in cold PBS/protease inhibitor (PI, Complete EDTA-free, Roche/Merck, Darmstadt, Germany) at a ratio of 25:1 using micro pestles and sonication. The samples were then centrifuged at 10.000 g at 4 °C for 10 min and the supernatant was transferred into a new tube. The protein content was determined using the RotiQuant solution (Karl Roth GmbH, Karlsruhe, Germany) following manufacturer’s instructions. The alkaline phosphatase activity was measured according to previously published protocols^11^. Briefly, 2 µg protein per sample were diluted in 100 µl of cold PBS/PI containing various concentrations (0, 1, 2, 5, and 10 mM referring to the end volume of 200 µl) of the TNAP-inhibitor levamisole, and finally 100 µl CSPD (Disodium 3-(4-methoxyspiro {1,2-dioxetane-3,2′-(5′-chloro)tricyclo [3.3.1.13,7]decan}-4-yl)phenyl phosphate) ready-to-use reagent (0.25 mM, Roche, Germany) were added. The reaction was performed in white 96-well-plates with a clear bottom at 37 °C for 5 min. For measuring the luminescence, which was resulting from the degradation of CSPD reagent by TNAP enzyme, the Orion II Microplate Luminometer (Berthold Detection Systems, Pforzheim, Germany) was used.

### Synthesis of TNAP-specific riboprobes and in situ hybridization (ISH) to detect TNAP mRNA

ISH was performed according to standard protocols^45,46^. Proteinase K incubation and NBT/BCIP staining times were adjusted to age of embryos and to investigated tissues. RNA probes were synthesized from cloned partial mRNA sequences of *alpl* using the DIG or FLU RNA Labeling Kit (Roche, Basel, Switzerland). We used an *alpl* cDNA fragment of 434 bp size to synthesize specific anti-sense RNA probes. Sense probes were synthesized as negative control for each anti-sense probe and were used under the same reaction conditions. Additional details on experimental set-up is given in the supplementary methods.

### Combined TNAP ISH and proliferating cell nuclear antigen (PCNA) immunohistochemistry on adult zebrafish brains

TNAP ISH was performed using the same TNAP-specific riboprobes as described for the embryonal stages subsequently followed by immunofluorescence (IF). PCNA-specific staining was performed using the primary antibody in a dilution of 1:200 (PCNA P8825, mouse monoclonal, Sigma-Aldrich, Antibody Registry nr. AB_477413) and anti-mouse IgG Alexa Fluor 488 (A-11001, Thermo Fisher Scientific, Antibody Registry nr. AB_2534069) secondary antibody in a dilution of 1:1,000. Finally, the brains were embedded using the JB-4 embedding kit (Polysciences, Warrington, PA, U.S.) according to manufacturer’s instruction and the brain samples were sectioned in 4 µm slices. Additional details on experimental set-up is given in the supplementary methods. Brain regions were sketched, identified and named according to Wullimann et al.^47^.

### Immunohistochemistry

Immunohistochemistry against acetylated α-Tubulin was performed according to previously published protocols^48^. The primary monoclonal mouse antibody IgG2b 6-11B-1 (sc-23950, Santa Cruz Biotechnology, Antibody Registry nr. AB_628409) was used in the dilution of 1:100. The anti-mouse IgG Alexa Fluor 488 (A-11001, Thermo Fisher Scientific, Antibody Registry nr. AB_2534069) secondary antibody was used in a dilution of 1:1,000 in PBST/Triton X-100/10% sheep serum (Sigma-Aldrich/Merck) including Hoechst 33,342 nuclear counterstain (dilution 1:10.000/PBST, Invitrogen).

### ELF 97 staining (AP-activity staining)

ELF 97 Phosphatase Substrate (Thermo Fisher Scientific), which is converted into a fluorescence precipitate by active AP enzymes, was used on adult brain cryosections of *AB/AB* wildtype fish, according to the manufacturer’s instructions with certain modifications. Specificity control of ELF staining was performed by TNAP inhibitor application and is shown in Fig. S15. Additional details on experimental set-up is given in the supplementary methods.

### Incubation of zebrafish embryos with TNAP inhibitors

The embryos were collected directly after fertilization, the chorion was punctured using a glass needle, and treatment with the respective inhibitors was performed in a final concentration of 0.5, 1, and 10 mM in 0.3× Danieau’s medium for levamisole (Merck, Darmstadt, Germany) and in a concentration of 10, 50, and 100 µM for MLS-0038949 (Merck, Darmstadt, Germany). Incubations were performed until 24 and 48 hpf for IF and 120 hpf for mineralized tissue and cartilage staining (change of medium after 48 h). For the MLS-0038949 treatment, DMSO served as a solvent control, in accordance with the volume of the highest MLS-0038949 concentration used (10 µl DMSO in 10 ml Danieau’s medium; final DMSO concentration: 14 mM). The chorion was punctured prior to treatment in order to guarantee a proper and reproducible incubation with the respective inhibitor.

### Alcian-blue and Alizarin-red double staining

Cartilage and mineralized structures were visualized by acid-free double staining with Alcian-blue and Alizarin-red according to previously published protocols^49^. Zebrafish were fixated with 4% PFA/PBS for 2–4 h and afterwards incubated with 50% EtOH for 10 min. Double staining was performed on a shaker overnight prior to washing with ddH_2_O. Afterwards, the samples were bleached for 20 min in 3% H_2_O_2_/PBS and subsequently washed with glycerol solutions containing KOH (20% glycerol in 1% KOH, 50% glycerol/1% KOH, and 50% glycerol/0.1% KOH (storage buffer)).

### Imaging

Depending on the experiment, images were acquired either with a Leica S8 APO Stereomicroscope (whole embryos), a Zeiss Imager A1 (ISH), or a Nikon A1 + Laser scanning confocal microscope (IF). In order to visualize the NBT/BCIP fluorescence signal on ISH sections, a corresponding near infrared filter was used^50^. To visualize ELF 97 staining a UV/DAPI longpass fluorescence filter set was used. Images were processed by using ImageJ/Fiji (https://fiji.sc/), CorelDraw Graphics Suite x8 software (Corel Corporation), and EnfuseGUI (https://software.bergmark.com/enfuseGUI/Main.html).

## Results

**Figure 1:**
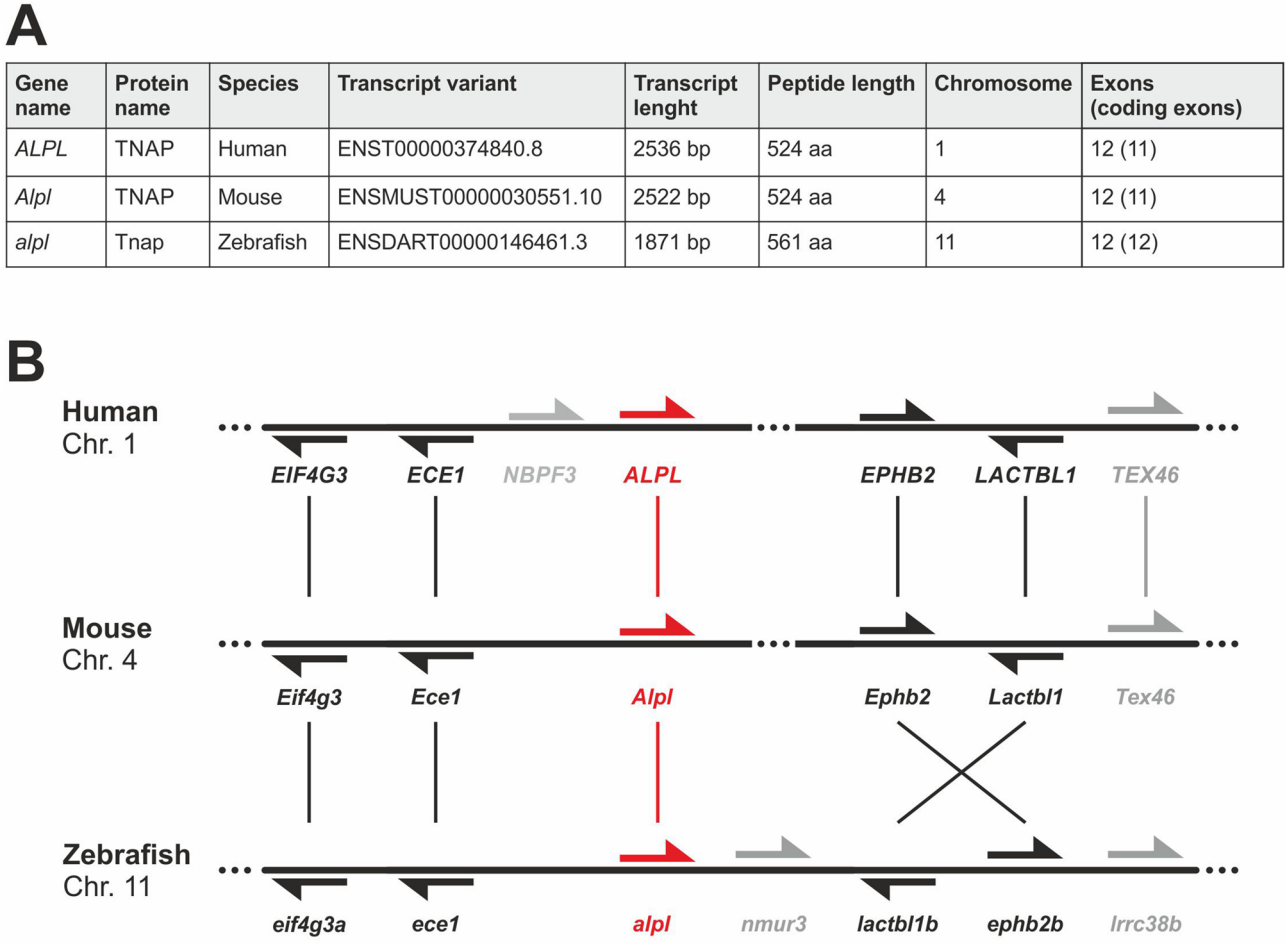
(**A**) Comparison of *alpl* orthologues between human, mouse and zebrafish. (**B**) Conservation of synteny between human, mouse, and zebrafish. Neighboring genes of the *ALPL* (Chr. 1), *Alpl* (Chr. 4), and *alpl* (Chr. 11) genes display similar order among the three organisms, which indicates a high level of conservation between species. The zebrafish *alpl* gene is not duplicated within the genome. The direction of the arrows indicates the orientation of the respective gene in the genome. Grey arrows and names indicate genes only detectable in single species. Genomic data was obtained from the ENSEMBL (genome assembly: GRCh38.p13, GRCm38.p6, GRCz11) and Genomicus v98.01 databases.

The *ALPL* gene encoding the ectoenzyme TNAP (tissue-nonspecific alkaline phosphatase) is widely conserved across a wide number of animal species (Treefam family: TF323513; ENSEMBL gene tree: ENSGT00950000183063). For providing consistent differentiation between species as well as between gene and protein, we used the respective conventional denominations in our manuscript. The gene name is *ALPL* in human, *Alpl* in mouse, and *alpl* in zebrafish, whereas the protein is called TNAP in human and mouse but Tnap in zebrafish. Detailed information on the human, murine, and zebrafish orthologues are listed in Fig. 1A.

Transcript alignment of the zebrafish *alpl* gene with the human and mouse sequences revealed an identity of 61% and 63%, respectively. In contrast to many other genes, the *alpl* gene is not duplicated within the zebrafish genome. Interestingly, the peptide alignment resulted in 67% overall identity to mouse and humans. Moreover, the alignment showed a remarkable high conservation level of 79% identity and 86% similarity in amino acid sequences of the zebrafish and the human alkaline phosphatase domain. Additional synteny analysis further revealed that genes located in close proximity to the respective *ALPL*, *Alpl*, and *alpl* locus were showing a high level of genetic conservation between human, mouse, and zebrafish on the respective chromosomes 1, 4, or 11 (Fig. 1B; Genomicus v98.01 database). Taken together, this in silico data shows genetic conservation between vertebrate species and implies retained Tnap/TNAP enzyme activity.

### Determination of the spatio-temporal expression pattern of *alpl* in zebrafish embryos

**Figure 2:**
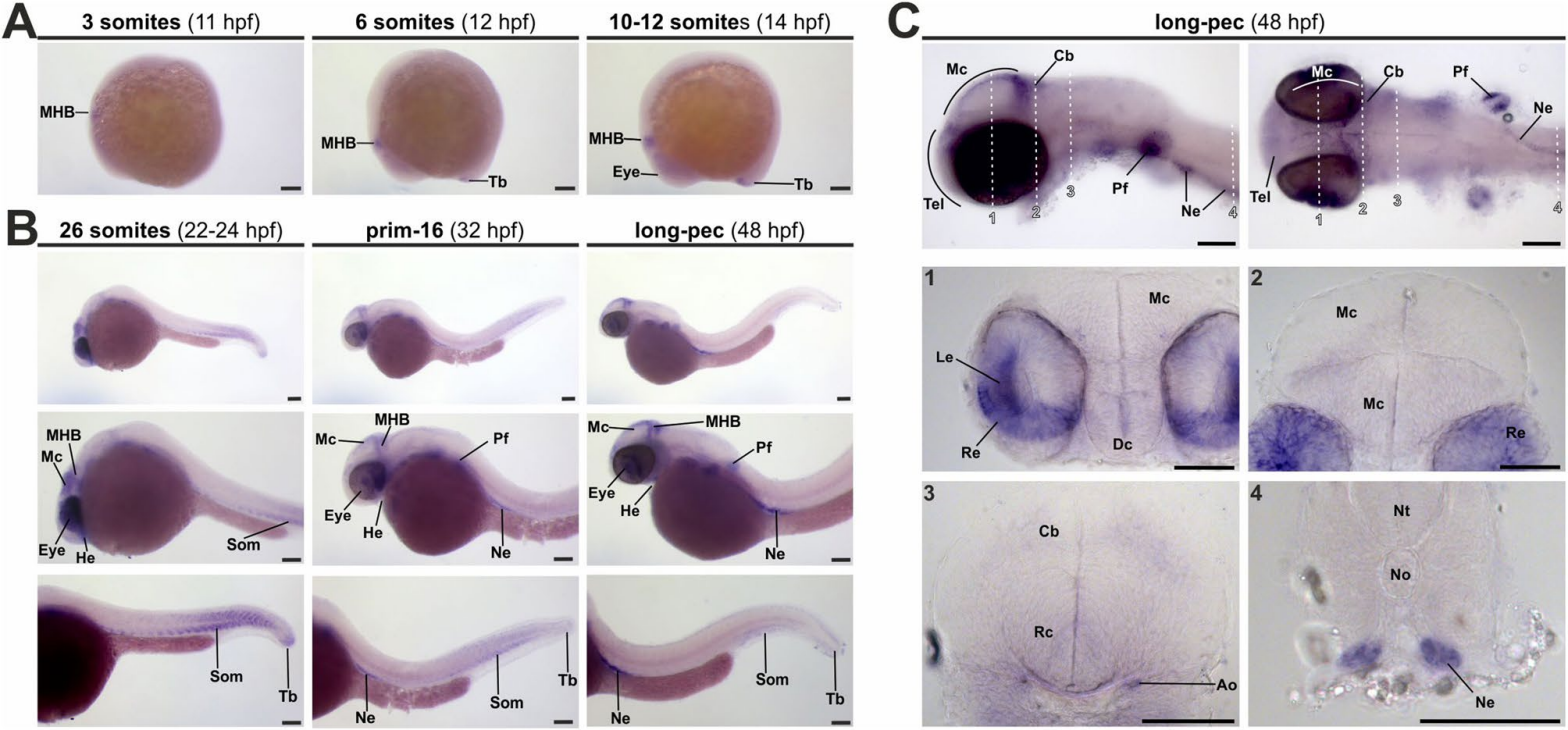
*alpl* expression during zebrafish embryogenesis. (**A**) and (**B**) Spatio-temporal expression pattern of *alpl* in zebrafish embryos at early segmentation (3–12-somites, A) until long-pec stages (48 hpf, B). (**C**) Detail images of head regions and of sections stained via ISH for *alpl* at long-pec stadium. Dashed white lines indicate level of sections shown at higher magnifications (1–4). Scale bar: 100 µm. Ao, dorsal aortic root; Cb, cerebellum; Dc, diencephalon; He, heart tube/heart; Le, lens; Mc, mesencephalon; MHB, midbrain-hindbrain boundary; Ne, nephros; No, notochord; Nt, neural tube; Pf, pectoral fin; Rc, rhombencephalon; Re, retina; Som, somites; Tb, tail bud.

In order to determine the spatio-temporal expression pattern of *alpl*, initial whole mount ISH with *alpl*-specific probes was performed on zebrafish embryos at different developmental stages (from 3 somites to 48 hpf; Fig. 2).

ISH showed the first *alpl* expression at early somitogenesis stages (11 hpf) within the midbrain-hindbrain boundary (MHB) region of the neural keel (Fig. 2A). During subsequent stages of zebrafish development, *alpl* expression was detected in more distinct areas: the MHB, the mesencephalon, the eyes (including retina and lens), the pectoral fin, the heart field, and the tail bud (Fig. 2B). The *alpl* expression in somites at later stages between 22 and 48 hpf was restricted to posterior parts of the trunk and was temporally tightly regulated (Fig. 2B; sense controls are shown in Fig. S2). For getting a more detailed impression of *alpl* expression within different tissues, sections of 48 hpf zebrafish embryos were analyzed (Fig. 2C). Low expression signals were detected in the developing neural tissues: diencephalon, optic tectum, as well as at the MHB. However, strong *alpl* expression was prevalent in the dorsal aortic root, the pectoral fin, the nephros, the retina, and the lens. In summary, expression analysis in the developing zebrafish embryo indicates tight regulation of *alpl* expression during embryonic stages and aligns with previously published expression patterns in other vertebrates (see Discussion section, Table 1).

### Analysis of endogenous *alpl* expression and Tnap-activity in adult zebrafish tissues

**Figure 3:**
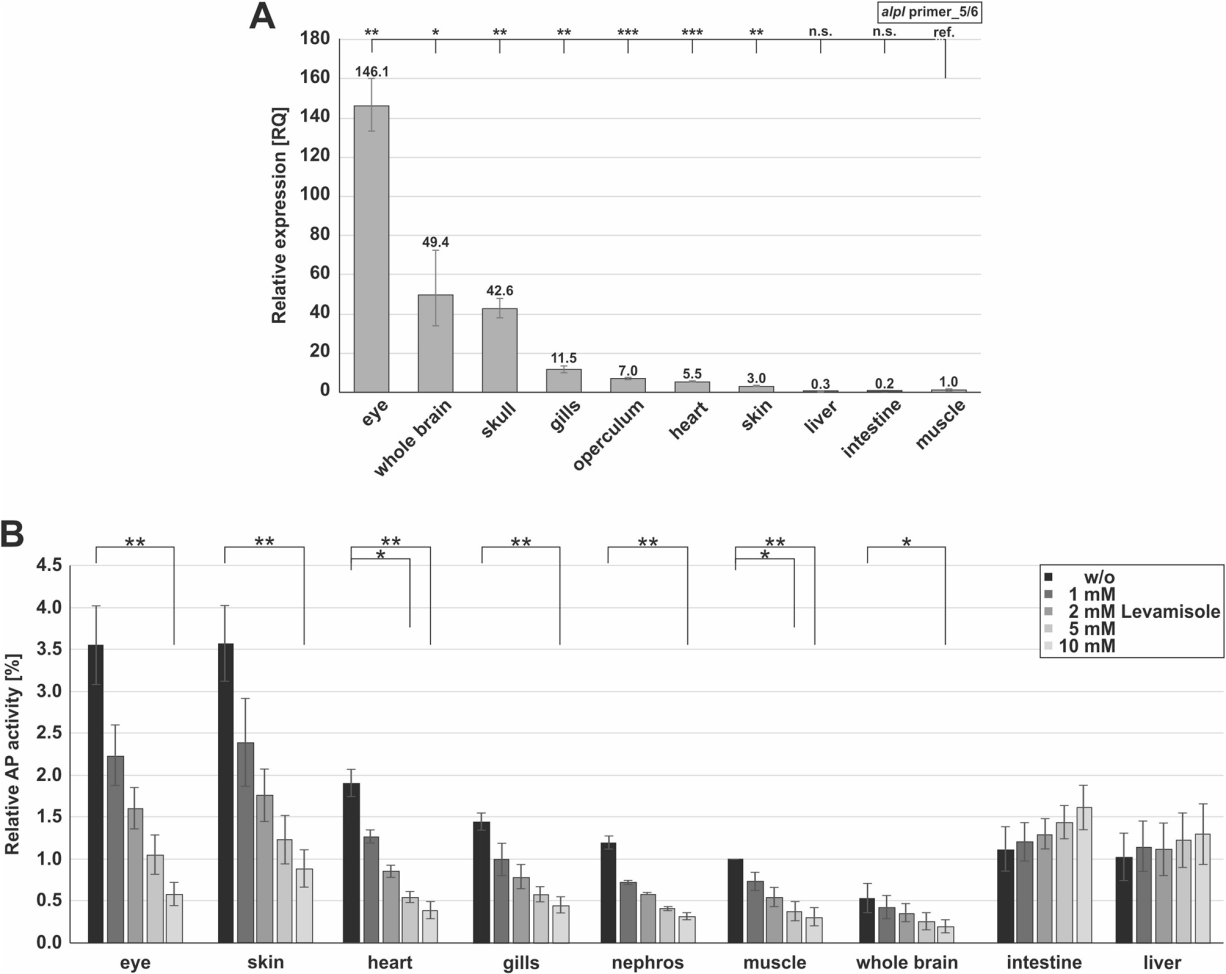
Expression pattern of *alpl* and activity of Tnap in adult zebrafish tissues. **(A**) *alpl* expression in different organs of adult zebrafish at the mRNA level determined with qPCR targeting exon 5/6. RQ: relative quotient (here calculated in comparison to the expression level of muscle samples). Statistics were calculated using two-sided student t-test, paired; ***p ≤ 0.001, **p < 0.01, *p < 0.05, n.s., not significant; ref, reference. **(B**) Enzymatic AP-activity in different organs of adult zebrafish measured with a CSPD-assay after treatment with different concentrations of the specific inhibitor levamisole. **p < 0.01, *p < 0.05. Normalization was performed using values for non-treated muscle samples. n = 3, technical replicates.

To determine the endogenous *alpl* expression and Tnap-activity in adult zebrafish, different organs of adult zebrafish were analyzed using qPCR (Fig. 3A) and AP-activity assay (CSPD-assay; Fig. 3B).

Highest qPCR relative quotient (RQ) values of *alpl* expression (normalized to the expression level of muscle samples) were detected within the eyes (RQ: 146.1), followed by the whole brain (RQ: 49.4), and the skull samples (RQ: 42.6; Fig. 3A). However, gills (RQ: 11.5), operculum (RQ: 7.0), heart (RQ: 5.5), and skin (RQ: 3.0) displayed low *alpl* expression, whereas liver and intestine samples did not show any detectable *alpl* expression at all. Additional qPCR experiments utilizing a second *alpl*-specific primer pair confirmed the expression data (Fig. S1).

The qPCR data was complemented with measurements of enzymatic Tnap-activity within several organs isolated from adult zebrafish (normalized to the activity levels in the non-treated muscle samples; Fig. 3B). The highest relative AP-activity (Fig. 3B, black bar, w/o levamisole) was detected in the skin (3.57) and the eyes (3.55). Additionally, a medium level of relative AP-activity was observed in the heart (1.91), in the nephros (1.19), and in the gills (1.44), directly followed by the intestine (1.12) and the liver (1.03). Low relative AP values were observed in whole brain samples (0.53) of adult zebrafish. In order to reveal the portion of Tnap-activity included in the total AP-activity, protein lysates were additionally treated with different concentrations of the Tnap-specific inhibitor levamisole, in a range between 1 and 10 mM. Interestingly, levamisole treatment was able to significantly decrease the AP-activity in all samples depending on the applied concentration, except for intestine and liver samples (Fig. 3B, dark to light grey bars). Comparing the mRNA expression level with the detected enzymatic activity, low levels of *alpl* mRNA but high Tnap-activity values were detected in skin samples, while high mRNA expression but low Tnap-activity were detected in whole brain samples. Apart from these, the *alpl* expression and Tnap-activity were corresponding to each other.

### Basic *alpl* expression and Tnap-activity analysis in adult zebrafish brain

**Figure 4:**
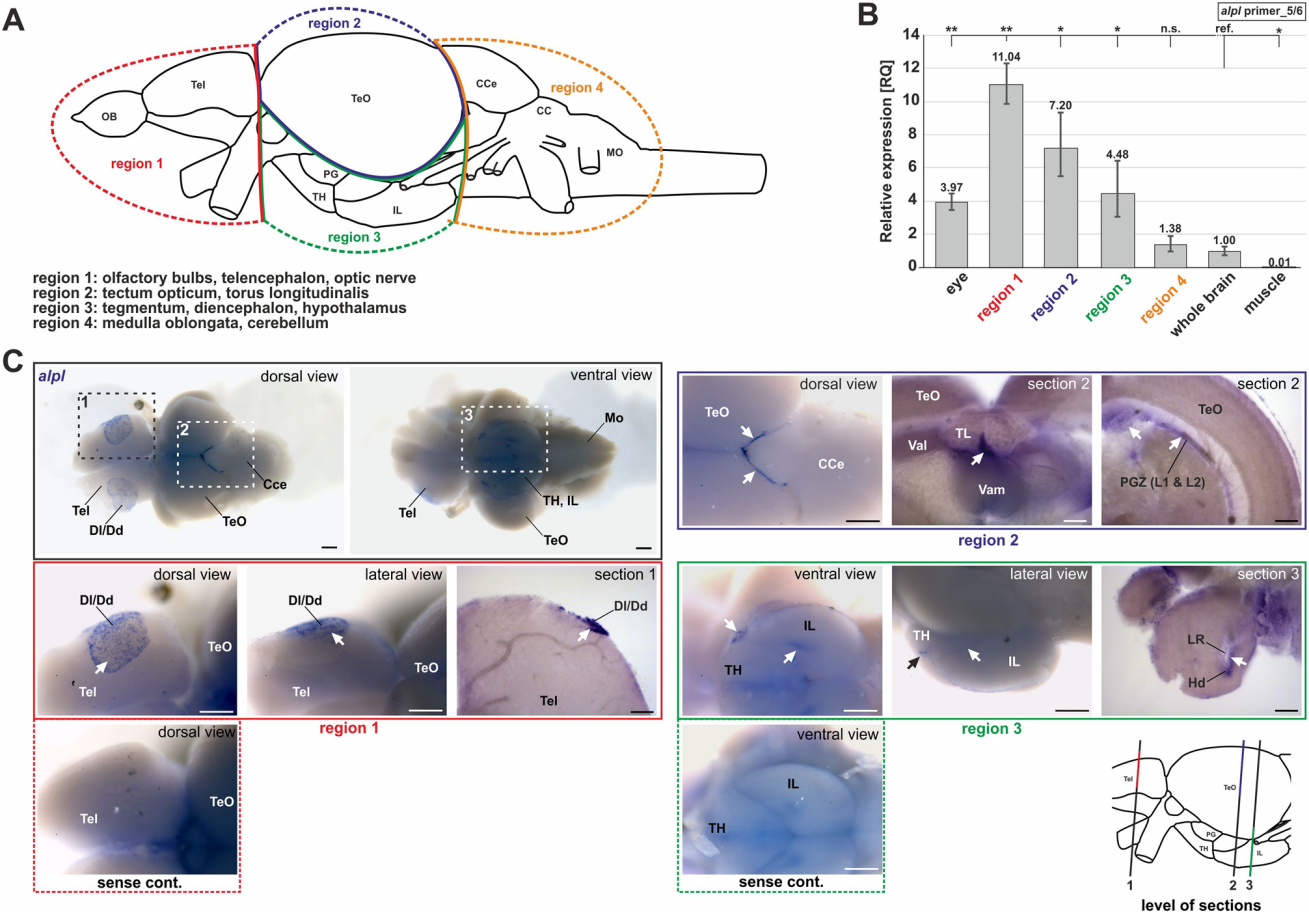
*alpl* expression pattern in whole adult zebrafish brains. (**A**) Scheme of different brain regions used for qPCR. (**B**) *alpl* expression at the mRNA levels determined by qPCR targeting exon 5/6 in different regions of adult zebrafish brain. Statistics were calculated using two-sided student t-test, paired; **p < 0.01, *p < 0.05**,** n.s., not significant; ref, reference. **(C**) ISH visualized expression domains of *alpl* in whole adult brains (marked by black and white arrows). Signals were detected in the telencephalon, tectum opticum and hypothalamus. Vibratome cross sections of single regions showed tissue-internal *alpl* expression. Control samples were hybridized with sense probe instead of antisense probe. Scale bar: 250 µm (whole brain images); 100 µm (close up view and sections). CCe, corpus cerebelli; Dl/Dd, lateral/dorsal zone of telencephalon; Hd, dorsal zone of the periventricular hypothalamus; IL, inferior lobe of hypothalamus; LR, lateral recess of diencephalic ventricle; Mo, medulla oblongata; PG, preglomerular area; PGZ, peripheral growth zone (layer 1 and 2); PiT, posterior inferotemporal cortex; Tel, telencephalon; TeO, tectum opticum; TH, tuberal hypothalamus; Val, ventral anterior thalamic nucleus; lateral part; Vam, ventral anterior thalamic nucleus; medial part.

Besides embryonic stages and whole brain samples, we analyzed the *alpl* expression pattern in distinct adult zebrafish brain regions by using qPCRs (Fig. 4B) and ISH (Fig. 4C).

For a detailed analysis of *alpl* expression, adult brains were dissected into four different regions, namely telencephalon (region 1: olfactory bulbs, telencephalon and otic nerve), cortex (region 2: tectum opticum and torus longitudinalis), ventral mesencephalon (region 3: tegmentum, diencephalon, hypothalamus), and rhombencephalon (region 4: medulla oblongata, cerebelum) as schematically depicted in Fig. 4A. The results of the respective qPCR analysis (RQ calculated in comparison to the expression levels of whole brain samples; cDNA levels normalized to the expression of *gapdh* and *eef1a1l1*; Fig. 4B) revealed the highest expression level of *alpl* in the telencephalon (RQ: 10.04), followed by the cortex (RQ: 7.20), the mesencephalon (RQ: 4.48), and finally the rhombencephalon (RQ: 1.38). Additionally, samples from eyes were analyzed as a positive control. Subsequently, ISH was performed on whole adult zebrafish brains (Fig. 4C). The *alpl* signals showed restricted expression in the telencephalon, the optic tectum, and the hypothalamus. Control samples treated with an *alpl* sense probe, did not reveal any specific signals within these regions. Aberrant signal was visible in the brain treated with the sense control, but these signals were strongly differing from the actual ISH signals and can therefore be considered as nonspecific and not interfering with the results of the ISH. Vibratome sections of the stained brains further clarified *alpl* expression in the dorsal diencephalon, the lateral recess, the ventral anterior thalamic nucleus, the hypothalamus, and the peripheral growth zone.

**Figure 5:**
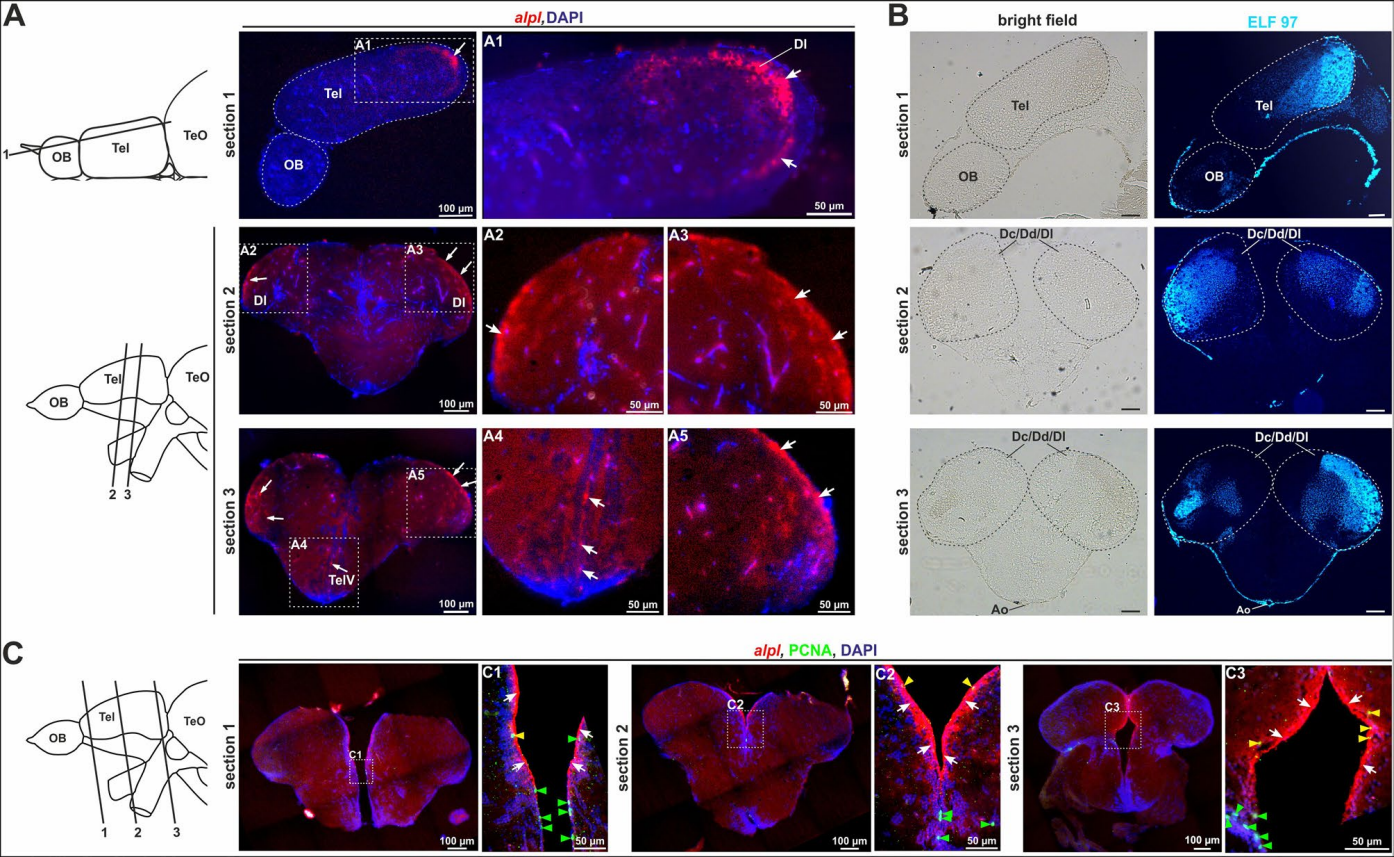
*alpl* expression pattern (combined with PCNA IF) and Tnap-activity in sections of the adult telencephalon. (**A**) Sagittal and cross sections of the telencephalic area of an adult zebrafish brain stained with *alpl* ISH and nuclear staining with DAPI. Signals were detected in the dorsolateral zone of the telencephalon (Dl) and in the ventricular zones, among others the telencephalic ventricle (TelV). The cutting planes of the different sections are indicated through the black lines and marked with numbers. Scale bars: 50 µm or 100 µm **(B**) ELF 97-staining showed sites of AP-activity within the telencephalon at sites of *alpl* expression. Scale bars: 100 µm. (**C**) Cross sections of the telencephalon of an adult zebrafish brain co-stained with *alpl* ISH (red) and IF for proliferation marker PCNA (green). Blue fluorescence depicts DAPI nuclear staining and gives structural information. C1, C2 and C3 are high resolution images of the respective regions of interest. The *alpl* ISH signals were indicated with white arrows, positive PCNA IF signals were marked with green arrowheads, and merged signals for both were highlighted using a yellow arrowheads. Scale bars: 50 µm or 100 µm. Ao, dorsal aortic root; Dc/Dd/Dl, central/dorsal/lateral zone of dorsal telencephalon; OB, olfactory bulb; Tel, telencephalon; TeO, Tectum opticum; TeIV, telencephalic ventricles.

Furthermore, we performed fluorescence analysis on JB-4 embedded plastic sections in order to increase ISH specificity and resolution of *alpl* expression analyses within the adult brain. Conventional NBT/BCIP *alpl* ISH signals were visualized by its near infrared fluorescence^50^ and sections were counterstained with DAPI as a nuclear marker (Fig. 5A). In sagittal sections, *alpl* expression was detectable in the dorsolateral zone of the telencephalon (Fig. 5A, magnification A1). The transverse sections confirmed *alpl* expression in the lateral zone of the dorsal telencephali (Dl; Fig. 5A, magnifications A2, A3 and A5) and in the ventricular zones, e.g. the telencephalic ventricle (TelV; Fig. 5A, magnification A4). To correlate *alpl* expression and the enzymatic function of Tnap within the adult zebrafish brain, we performed ELF^T^ 97 staining on corresponding telencephalon cryosections (Fig. 5B). Strong Tnap-activity could be visualized broadly within the telencephalon in the central, dorsal and lateral dorsal zones (Dc/Dd/Dl). Subsequently, adult zebrafish brains marked for *alpl* expression were co-stained for PCNA via ISH/IF. Transverse sections were prepared at three different levels within the telencephalon and revealed *alpl* expression in close proximity or co-localized to PCNA positive cells (Fig. 5C, magnifications C1 to C3, green arrowheads). Moreover, double positive cells were detected at the border of the medial zone of the telencephalon (yellow arrowheads).

**Figure 6:**
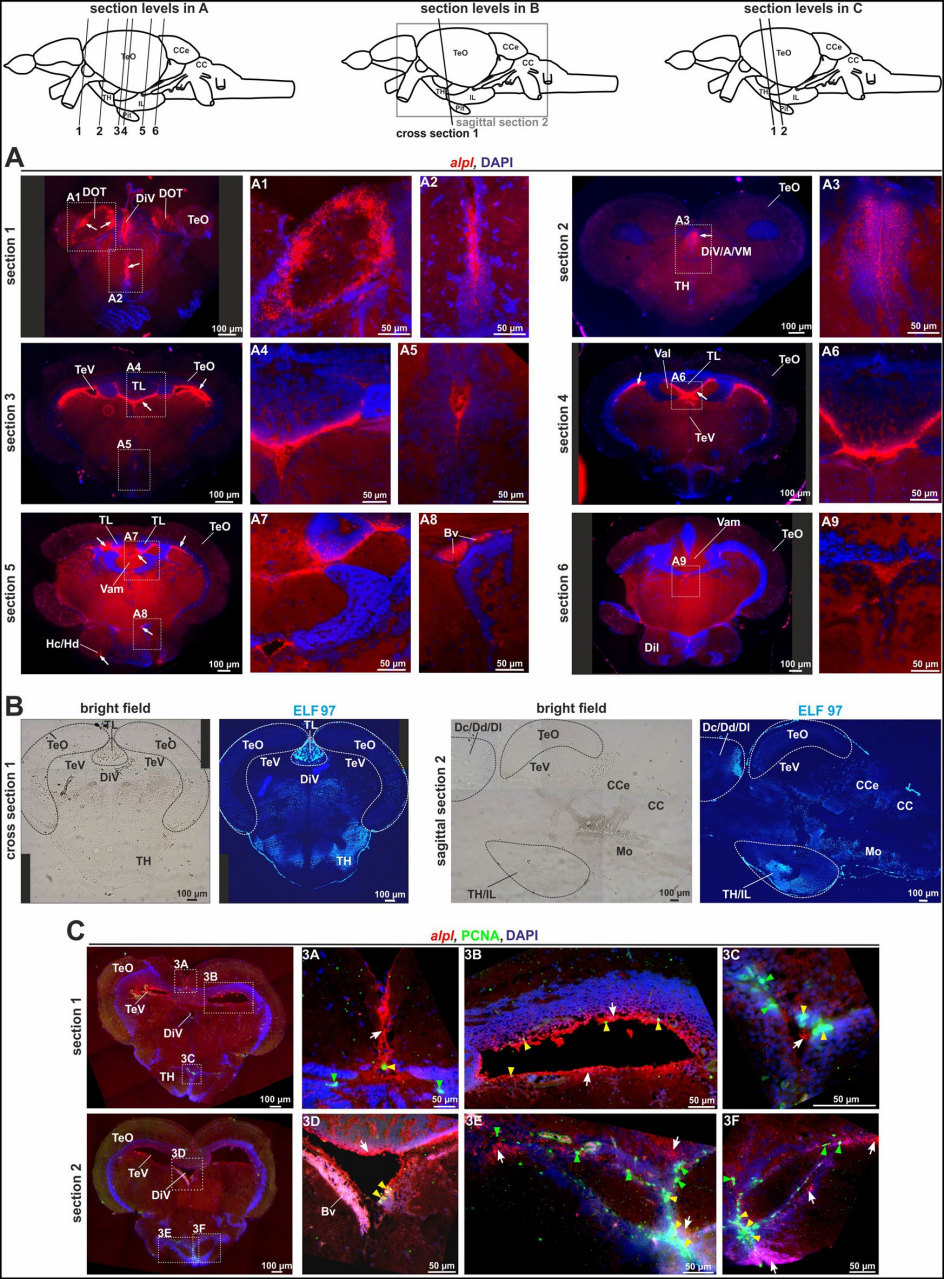
*alpl* expression pattern (combined with PCNA IF) and Tnap-activity in sections of the adult mesencephalon, diencephalon, and hypothalamus. The cutting planes of the different sections were indicated in schematic overviews through black lines and marked with numbers. (**A**) Cross sections of adult zebrafish brain stained with *alpl* ISH (red) and nuclear staining using DAPI (blue) in the region of the mesencephalon, diencephalon, and hypothalamus. Scale bars: 50 µm (high resolution photos) or 100 µm (overview). The *alpl* ISH signals were detected, among others, in ventricular areas (DiV, TeV), the dorsomedial optic tract (DOT), and the hypothalamus (HC). (**B**) ELF 97-staining showed sites of AP-activity within the torus longitudinalis (TL), the hypothalamus (TH/IL), and the medulla oblongata (Mo). (**C**) Cross sections of adult zebrafish brain co-stained with *alpl* ISH (red), PCNA IF (green), and DAPI (blue) in the region of the mesencephalon. Scale bars: 50 µm or 100 µm. Merged *alpl* ISH, PCNA IF, and DAPI signals were shown. The respective localizations of the region of interests, depicted in the high resolution pictures, was indicated with white boxes. The *alpl* ISH signals were marked with a white arrows, PCNA signals with a green arrowheads, and cells that were positive for *alpl* and PCNA were indicated with yellow arrowheads. Bv, blood vessel; CC/CCe, cerebellum; Dc/Dd/Dl, central/dorsal/lateral zone of telencephalon; Dil, inferior lobe; DiV, diencephalic ventricle; DOT, dorsomedial optic tract; Hc/Hd, caudal/dorsal zone of periventricular hypothalamus; Mo, Medulla oblongata; TeV, telencephalic ventricles; TeO, Tectum opticum; TH/IL, hypothalamus; TL, torus longitudinalis; A/VM, anterior/ ventromedial thalamic nucleus; Val/Vam, valvula cerebelli.

Additional JB-4 sections were prepared at different levels of the mesencephalon, a second brain region showing high *alpl* expression (Fig. 6). Six different cutting planes in the area, including the mesencephalon, diencephalon, and hypothalamus were initially investigated (Fig. 6A) and implied that *alpl* expression is predominantly prevalent in the ventricular areas (DiV, TeV; Fig. 6A, magnifications A2, A3, A5 and A9), the valvula cerebelli/torus longitudinalis (Vam/TL, Fig. 6A, magnifications A4, A6 and A7), the dorsomedial optic tract (DOT; Fig. 6A, magnification A1), the caudal hypothalamus (HC; section 5), and in blood vessels (Bv, Fig. 6A, magnification A8). ELF 97 staining was again performed to visualize different areas of AP activity (Fig. 6B), most prominently within the torus longitudinalis (TL), the hypothalamus (TH/IL), and in single regions of the medulla oblongata (MO). Combined *alpl* ISH and PCNA IF confirmed that *alpl* was expressed in cells of the ventricular regions, either within or surrounding PCNA positive cells (Fig. 6C; magnifications 3A–3F). In the DiV, the PCNA expressing cells were located very closely to the *alpl* positive cells (Fig. 6C, magnifications 3A and 3D) and sometimes showed overlapping patterns. More cells that expressed both, *alpl* and PCNA, could be detected besides the DiV also in the area between the tectum opticum (TeO) and the commissura tecti (Ctec; Fig. 6C, magnifications 3B and 3E). A high number of PCNA positive cells were located in the lateral recess (LR), a ventricle embedded in the hypothalamus (TH/IL; Fig. 6C, magnifications 3C and 3F). Close to this specific region, further *alpl* positive cells were detectable. In addition, analysis of the rhombencephalon was analogously performed, but only weak *alpl* expression was detected by ISH in this area (data not shown).

In summary, the analysis of the *alpl* expression and Tnap-activity pattern in the adult zebrafish brain revealed, that the highest prevalence of Tnap is localized to the telencephalon, here especially to the dorsal and lateral regions. Additionally, *alpl* expression is, among others, prevalent in the hypothalamus, the thalamus, and interestingly also in cells of the ventricular regions, which are important zones for adult neurogenesis. Our own, novel data have been compared to previously published expression patterns in primates, mice and zebrafish in Table 1 in the discussion section.

### Relevance of Tnap in the early development of the nervous system and the skeletal structure of the skull

**Figure 7:**
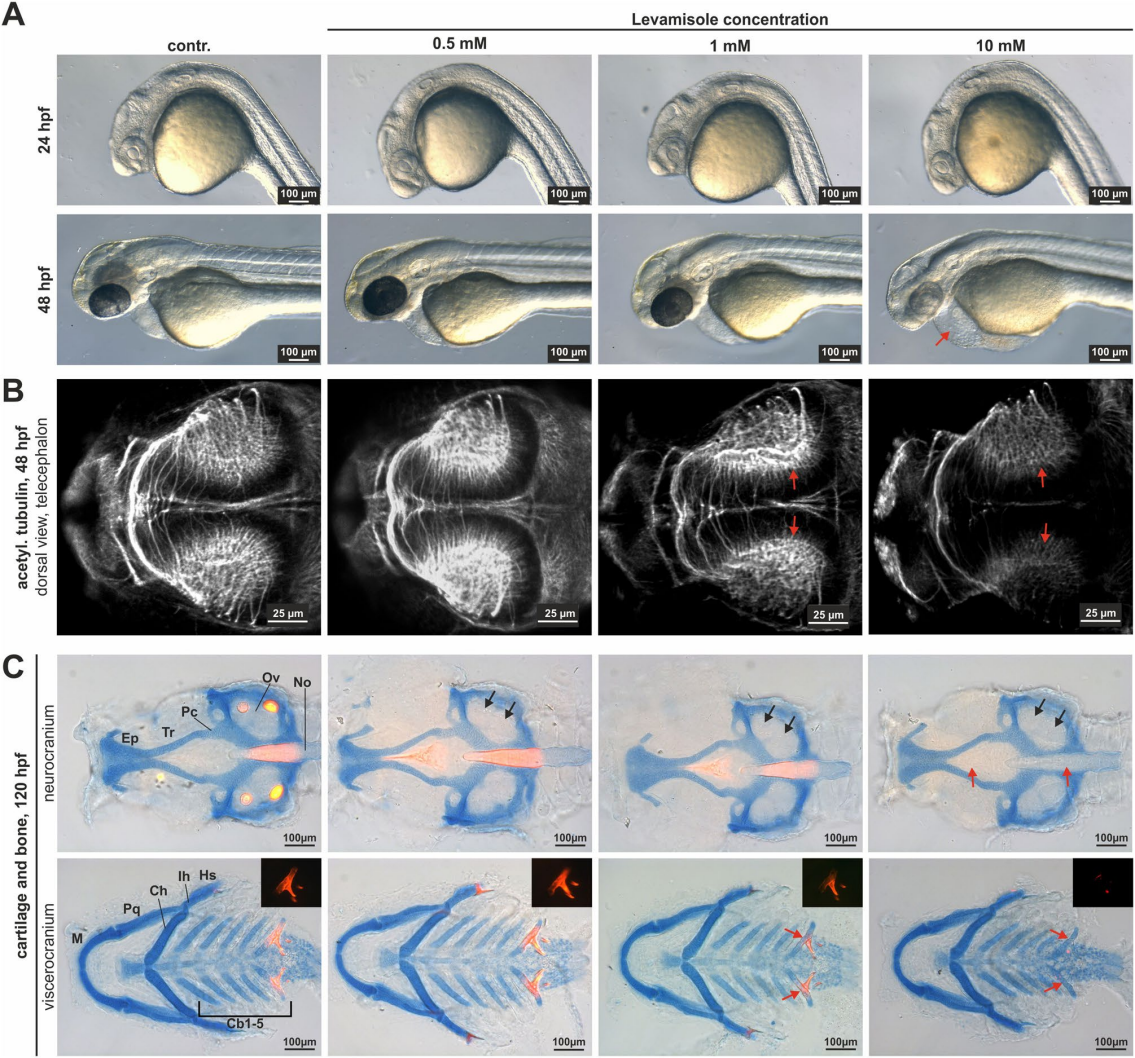
Inhibition of Tnap-activity leads to alterations in axon and mineralization. (**A**) Incubation of zebrafish embryos in different concentrations of levamisole altered normal development at 24 and 48 hpf depending on its concentration and resulted in cardiovascular defects (red arrow indicates enlarged pericardial sac). (**B**) Investigation of axonal growth by acetylated tubulin staining detected reduced growth within the optic tectum at 48 hpf. Scale bars: 25 µm. Red arrows point to differences in neural development under levamisole treatment. (**C**) Blocking of Tnap-activity resulted in hampered mineralization and influenced craniofacial development at 120 hpf. Alcian-blue stains the cartilage and Alizarin-red the mineralized structures. Fluorescence signals of Alizarin-red are additionally depicted in the upper right corner. Scale bars: 100 µm. Black arrows display the morphological changes of the cartilage and red arrows the missing mineralized structures. Cb, ceratobranchial cartilage; Ch, ceratohylal cartilage; Ep, ethmoid plate; Hs, hysosympletic; Ih, interhylal; M, Meckel’s cartilage; No, notochord; Ov, otic vesicle; Pc, parachordal cartilage; Pq, palatoquadrate cartilage; Tr, trabecular cartilage.

In order to investigate a possible influence of Tnap inhibition on the early development of the nervous system and the skeleton in zebrafish, embryos were treated with the Tnap-specific inhibitor levamisole at different concentrations (0.5, 1 and 10 mM). After morphological analysis of the embryos within the first 48 h (Fig. 7A), functional consequences of decreased Tnap-activity on early nervous system development were analyzed. Axonal outgrowth within the optic tectum was visualized by IF staining for acetylated tubulin at 48 hpf and investigated with a confocal microscope (Fig. 7B). Moreover, the craniofacial cartilage and mineralization progress of levamisole treated embryos was analyzed at 120 hpf, using combined cartilage and mineralized tissue staining (Alcian-blue and Alizarin-red; Fig. 7C).

Levamisole at low doses (0.5 and 1 mM) had no or only a slight impact on the regular morphological development of the treated zebrafish embryos at 24 and 48 hpf. Abnormal developmental effects, aberrations to untreated/DMSO control embryos and increased lethality were concentration dependent and increased in intensity with higher levamisole concentration (Fig. 7A; data not shown). At 10 mM, altered rates of blood flow, enlarged pericardial sacs, impaired normal heart, and vascular development were observed in the majority of 48 hpf embryos investigated (red arrow in Fig. 7A). The analysis of axonal growth after levamisole treatment (48 hpf) indicated aberrant axonal outgrowth of neurites in the optic tectum even at a low concentration of 1 mM levamisole (marked with red arrows; Fig. 7B). In General, increasing the levamisole concentrations led to more pronounced effects on the axonal outgrowth.

Furthermore, cartilage and mineralized tissue staining of levamisole treated embryos revealed aberrant craniofacial development (see Fig. 7C). Interestingly, especially mineralized structures were deteriorated in embryos treated with 1 mM and were completely absent in embryos treated with 10 mM levamisole (120 hpf; Fig. 7C, red arrows). The respective control embryos and the embryos treated with the lowest inhibitor concentration of 0.5 mM levamisole developed normal mineralized structures at this developmental stage. Simultaneous cartilage staining with Alcian-blue revealed morphological changes of the developing skull structure, which were statistically evaluated for preparations of the viscero- and neurocranium (Figs. S5 and S6; n = 10 for each group). Most significant changes were detected in measurements of branchial arch and anterior–posterior length within embryos treated with 10 mM levamisole. Treatment of zebrafish embryos with the alternative Tnap inhibitor MLS-0038949 showed very similar results for the development of the nervous system and the cartilage/mineralized tissue, what further confirmed the levamisole results (Fig. S13 and Fig. S14).

Zebrafish embryos were additionally microinjected with *alpl* Splice and ATG Morpholinos for knockdown experiments and for recapitulating the indicated influence of Tnap during the development of the nervous system and mineralization of the zebrafish (Fig. S3 and Fig. S4). Both Morpholinos led, analogous to the effects of the inhibitors, to distinct morphological changes depending on the respective concentration, e.g. altered rates of blood flow and an enlarged pericardial sac (Fig. S4A). Acetylated tubulin IF staining confirmed aberrant axonal growth in the optic tectum of *alpl* morphants at 48 hpf (Fig. S4B). The analysis of the craniofacial structures at 120 hpf confirmed morphological changes of cartilage structures and a missing development of mineralized structures after *alpl* knockdown (Figs. S4C). The changes of the cartilage structures were statistically evaluated, analogous to the analysis of levamisole treatment (Fig. S7, S8, S9, and S10; n = 10 for each group) and were compared to control Morpholino injected embryos (Figs. S11 and S12). Most significant changes were again detectable in measurements focusing on branchial arch and anterior–posterior length, but also measurements of the skull width were significantly altered. Additionally, mineralization of teeth, operculum, and cleithrum was limited in embryos treated with Tnap inhibitors and Morpholinos at 5 dpf (Fig. S14).

Taken together, our data reveals that zebrafish *alpl* expression and Tnap-activity can be detected during zebrafish embryonic development, in different adult organs, and in distinct adult brain regions. Tnap inhibition and *alpl* knockdown result in specific and comparable developmental defects, e.g. interference with axonal growth and mineralization. Moreover, the presented data is in accordance with descriptive and functional observations made in other vertebrates and therefore implies conservation of Tnap function also in zebrafish.

## Discussion

Although the molecular mechanisms of HPP-related deficits in bone formation and mineralization offer plausible explanations for the predominant skeletal phenotype, the molecular reasons behind a considerable number of HPP symptoms are to date not fully understood. These include the development of craniosynostosis in an actually repressive environment for mineralization processes and the increased risk for depression and/or anxiety disorders in HPP patients^51,52^. The high level of genetic conservation of the zebrafish *alpl* compared to other vertebrate orthologues, like human *ALPL*, implies that zebrafish might be suitable for functional analyses within bone and nervous system development^20,21,26^. Our presented zebrafish study now provides initial data on *alpl* expression patterns, describes functional consequences of Tnap inhibition during embryogenesis, and sets the scene for the establishment of a novel animal model in order to study HPP.

Initially, we investigated the endogenous expression of the *alpl* gene and of Tnap’s biochemical function using qPCR, AP-activity assays as well as whole mount ISH in embryonal stages and in adult zebrafish. During early segmentation stages of embryo development, *alpl* mRNA is predominantly detected in the MHB, the precursor structures of the eyes, and in the tail bud. In subsequent developmental stages until 48 dpf, *alpl* is expressed in the MHB, the diencephalon, the mesencephalon, the lens, the retina, the heart, the nephros, the tail bud, the somites, and the pectoral fin. Apparent expression pattern changes can be observed within somites over time, which implies tight regulation of *alpl* expression during embryogenesis. Interestingly, the pectoral fin is one of the earliest developing skeletal structures expressing *alpl* and precedes many other bony structures. Therefore, the detection of the *alpl*-specific signals in this precursor tissue might foreshadow its conserved role in regulation of the phosphate metabolism during mineralization in extremities^7^.

Furthermore, our experiments affirm that *alpl* mRNA and Tnap-activity are detectable in almost all analyzed adult tissues, apart from the intestine and the liver, which do not respond to the Tnap-specific inhibitor levamisole. The lack of responsiveness to levamisole leads to the assumption that those organs express an intestinal (*alpi.1* or *alpi.2*) instead of the tissue-nonspecific isoform of the AP (*alpl*). Additionally, the highest *alpl* expression levels along with strong AP-activity can be observed in the eyes, more precisely in the lens and the retina, which seems to be conserved between different vertebrate species and is in line with previously published literature^15^. However, no specific symptoms concerning the visual performance of HPP patients have been described so far and the exact role of TNAP/Tnap in the visual system and potential compensatory mechanisms remain to be unraveled in future experiments.

For detailed investigation of the nervous system related symptoms of HPP and for confirmation of qPCR and CSPD-assay results, the endogenous expression of *alpl* was additionally analyzed in dissected and in sections of adult zebrafish brains. The results show that *alpl* expression is prevalent in the telencephalon (especially in the dorsal and lateral telencephalic area), the tectum opticum, and in the hypothalamus (lateral recess, ventral anterior thalamic nucleus, peripheral growth zone). Moreover, we can frequently detect *alpl* expression in ventricular zones of the brain (e.g. TeIV and TeV). The *alpl* expression pattern implies restricted Tnap-activity in the zebrafish brain, but staining for ELF 97 shows rather wide areas of Tnap-activity in the telencephalon, in the hypothalamus, and in the torus longitudinalis. Proteolytic shedding processes might lead to cleavage and spreading of active Tnap enzyme within brain compartments and subsequently result in tissue-wide activity. Release of TNAP from the cellular membrane via degradation of its phospholipase mediated GPI-anchor has already been reported and might be one molecular mechanism for this observation^53,54^. Nevertheless, it is unclear to what extent those processes might lead to the observed discrepancy between the different staining methods. Another interesting observation are strong ISH signals within the dorsomedial optic tract (DOT), a white matter structure comprising predominantly of retinal ganglion cell axons. Localization of TNAP to axons of cultured hippocampal neurons has been previously reported and TNAP-activity is crucial for correct axonal growth by ATP degradation^10^. Another explanation for the discrepancy between ISH and ELF 97 data may further result from different sensitivity thresholds of the respective staining method.

The proliferation marker PCNA is partly co-localized or localized in close proximity to the *alpl* expressing domains in the brain sections. In total, 16 proliferative zones have already been described in the adult zebrafish brain, which are predominantly located at the ventricular surface, and incorporate neuronal stem cells^55^. Compared to zebrafish, the mammalian brain shows only limited capability of adult neurogenesis. Novel formation of neurons in adult brains is only possible in the subventricular zone (SVZ) of the lateral ventricles and the subgranular zone of the hippocampal dentate gyrus in humans^56^. Interestingly, Tnap-activity is indeed located in the ventricular and SVZ of mice including zones of adult neurogenesis^57^.

Prominent *ALPL* signals can be verified in specific brain regions of different mammals and in layers across the neocortex (occipital-, frontal-, and temporal lobe), for example in layer 4 of the primary visual and somatosensory cortices. Our zebrafish results are comparable to expression patterns observed in mammals and indicate that *alpl* transcripts can not only be detected during embryogenesis, but also in specific brain structures of adult animals. Generally, *ALPL* expression levels are higher during vertebrate development compared to mature organs, tissues (e.g. cerebral neuronal networks), or the complete animal^12,13,18,58^. The results of our own study and literature data on the *ALPL*, *Alpl*, and *alpl* expression in adult brains are summarized in the Table 1.

**Table 1:** Expression pattern of *ALPL*, *Alpl*, and *alpl* in adult brains of primates, mouse, and zebrafish.

Tissue	Primates	Mouse	Zebrafish
Blood vessels	+	+	+
Retina	+	+	+
*Forebrain*			
Diencephalon (hypothalamus, thalamus)	n.a	+	+
Telencephalon (amygdala, olfactory bulb, cortex, hippocampus, septum, pallium)	+	+	+
Olfactory bulb	n.a	+	n.a
*Midbrain*			
Ventricular zones (e.g. DiV and TeV in the midbrain)	+	+	+
Septum	n.a	+	+
*Hindbrain*			
Medulla oblongata, metencephalon	n.a	+	+
**References**	^6,13–15,59–62^	^15,16,18,57,60,63,64^ MGI database: 87,983	^15^ own data

n.a.: not available.

The expression of *alpl* and inhibition of TNAP-activity at very early embryonic stages of the zebrafish implies that the enzyme plays an important role during initial steps of development. This assumption is further promoted by patients suffering from genetically determined severe TNAP deficiency and by homozygous *Alpl* knockout mice, which often die very shortly after birth^65,66^. The inhibition of Tnap-activity, either with chemical components (levamisole and MLS-0038949) or by knockdown of *alpl* expression using Morpholinos, impairs the development of cartilage/bone as well as the nervous system in zebrafish^67^. Considering the known side effects of levamisole, like ion channel inhibition^68^, the investigation of MLS-0038949 and *alpl* knockdown was indispensable in order to confirm the results. Our studies with the alternative inhibitor MLS-0038949 and with two *alpl* Morpholinos affirm the levamisole results to the greatest extent. However, we observe differences between the two inhibitors, as MLS-0038949 is effective at much lower doses and displays a stronger effect on axonal growth even at 10 µM. Levamisole needs to be prevalent in higher doses for effective blocking and shows more prominent effects on bone development over time. Our results clearly show that both inhibitors and Morpholino knockdown are able to repress Tnap-activity, but differ in their influence on neuronal and skeletal development. These results imply different modes of TNAP functional reduction and might point to different biochemical consequences. TNAP/Tnap’s promoting role in hydroxyapatite crystallization is mediated by diminishing the amount of prevalent mineralization inhibitor PP_i_ in the extracellular space during in vitro osteogenic differentiation^69^. While axonal outgrowth is initiated by preventing the activation of the purinergic receptor of P_2_X_7_^7,10^. Overall, the observed effects of Tnap inhibition on early neuronal and bone development indicate a rather broad spectrum of Tnap efficacy, including effects on dephosphorylation of general substrates, such as PPi, ATP/ADP/AMP, and phosphorylated osteopontin^1,9,70^. Despite the low level of interference of the Splice Morpholino with the wildtype gene product and general concerns with Morpholino usage, the data are supporting our results gained with the Tnap inhibitors levamisole and MLS-0038949. However, we have to point out that, deviant of the Stanier et al. guidelines, no mutant lines have been generated yet, which would be essential for a complete characterization of the phenotype. The establishment of transgenic zebrafish lines has to be done in future research projects. Nevertheless, we think that we can trust the inhibitor and Morpholino data as they are confirming an influence of decreased Tnap-activity on mineralization processes and neuronal development, which is additionally going along with the phenotype of HPP patients.

In summary, our study identifies the zebrafish as a suitable model for the rare disease HPP. The analysis of endogenous *alpl* expression in zebrafish as well as first experiments on functional consequences of decreased *alpl* expression/Tnap inhibition on the embryonic development of bones and the nervous system pave the way for consecutive analysis of transgenic zebrafish lines in the future. These will help to further elucidate so far unsolved molecular mechanisms that are underlying certain HPP symptoms. One unsolved symptom to be investigated is how the paradox situation of premature suture fusion in the developing skull can be reconciled with an environment of hypomineralization in the rest of the body. Moreover, future studies have to investigate how neurological symptoms, like depression or anxiety disorders, can indeed robustly be linked to *alpl* deficiency in single brain regions or to specific neurons^51,71,72^. An important role of TNAP/Tnap during the processes of synaptogenesis, myelination, neurotransmitter metabolism, and axonal outgrowth has already been proclaimed, but has not been investigated in zebrafish^8,10,12,13^. Therefore, those cellular developmental functions definitely have to be considered for establishing a complete picture of TNAP’s function in the nervous system of zebrafish by applying cell-type-specific methods. Generally, our results of the embryonic and adult *alpl* expression analysis in zebrafish suggest a possible correlation to the pathology of the rare disease HPP and open up new perspectives concerning the neurological consequences of TNAP/Tnap dysfunctions.

## Supplementary information

Supplementary file1
