# Supplementary material for: Investigation of alpl expression and Tnap-activity in zebrafish implies conserved functions during skeletal and neuronal development
Source: Sci Rep. 2020 Aug 7;10:13321. doi: 10.1038/s41598-020-70152-5 (PMC7414108; doi:10.1038/s41598-020-70152-5)
Supplement: Supplementary file 1 — Supplementary file1 [file 41598_2020_70152_MOESM1_ESM.docx]

**Supplementary Information**

**Investigation of *alpl* expression and Tnap-activity in zebrafish implies conserved functions during skeletal and neuronal development.**

Barbara Ohlebusch^+^, Angela Borst^+^, Tina Frankenbach, Eva Klopocki, Franz Jakob, Daniel Liedtke*, Stephanie Graser*

* Both authors contributed equally to this work

^+^ Both authors contributed equally to this work

**Table S1:** Primers used in this study.

| Name | Primer sequence | Purpose |
| --- | --- | --- |
| zf_alpl_5/6_qPCR_for | TTCCTCTGCGGTGTCAAAGCCAA | qPCR, intron-spanning, located in exon 5, *alpl* (GRCz11: ENSDARG00000015546) |
| zf_alpl_5/6_qPCR_rev | AAGCAGCACTCGGGGTGGCAT | qPCR, intron-spanning, located in exon 6, *alpl* (GRCz11: ENSDARG00000015546) |
| zf_alpl_6/7_qPCR_for | CACAACAACGCGAGTGAACC | qPCR, intron-spanning, located in exon 6, *alpl* (GRCz11: ENSDARG00000015546) |
| zf_alpl_6/7_qPCR_rev | GGGGTACATGCTTCTCCGTC | qPCR, intron-spanning, located in exon 7, *alpl* (GRCz11: ENSDARG00000015546) |
| ef1a1-f01 | GCCCCTGGACACAGAGACTTCATCA | qPCR, intron spanning from exon 3 to 4; housekeeping reference, *eef1a1l1* (GRCz10: ENSDARG00000020850) |
| ef1a1-r01 | AAGGGGGCTCGGTGGAGTCCAT | qPCR, intron spanning from exon 3 to 4; housekeeping reference, *eef1a1l1* (GRCz10: ENSDARG00000020850) |
| Zf_gapdh_qPCR_fwd1 | GTGGAGTCTACTGGTGTCTTC | qPCR, intron spanning from exon 5 to 7; housekeeping reference, *gapdh* (GRCz10: ENSDART00000063800) |
| ZF_gapdh_qPCR_rev1 | GTGCAGGAGGCATTGCTTACA | qPCR, intron spanning from exon 5 to 7; housekeeping reference, *gapdh* (GRCz10: ENSDART00000063800) |
| Zf_TNAP_ribo_fwd1 | GACCGGGCCATTACTAGAGC | cloning, ISH probe #1, *alpl* exon 10 (Transcript; ENSDART00000146461.3) |
| Zf_TNAP_ribo_rev1 | TTTGTCCGGCAGTGGTCTTT | cloning, ISH probe #1, *alpl* exon 12 (Transcript; ENSDART00000146461.3) |
| Zf_TNAP_ribo_fwd2 | AATCCGTAAACGCAGGCTGA | cloning, ISH probe #2, *alpl* exon 2 (Transcript; ENSDART00000146461.3) |
| Zf_TNAP_ribo_rev2 | GCGTTGTTGTGACGATTCCC | cloning, ISH probe #2, *alpl* exon 6 (Transcript; ENSDART00000146461.3) |

**Supplementary Methods**

**RNA isolation, reverse transcription, and qPCRs**

Whole organs of adult fish and specific parts of adult brains were extracted and instantly frozen in liquid nitrogen. Samples were stored at -80° C until preparation. One ml QIazol Lysis reagent (Qiagen, Hilden, Germany) was added prior to homogenization using an Ultrathurax under constant cooling on ice. The sample in QIazol was incubated for 5 min at RT and centrifuged for 10 min at 12.000 g and 4° C and phenol/chloroform extraction was performed after standard protocols. Briefly, the supernatant was transferred into a new tube and 200 µl chloroform was added. The solution was vigorously mixed, incubated for 10 min at RT and centrifuged for 15 min at 12.000 g and 4° C. The aqueous phase was transferred into a new tube, 500 µl isopropanol was added, the solution incubated on ice for 15 min and afterwards again centrifuged for 10 min at 12.000 g and 4° C. The supernatant was discarded and the pellet washed with 1 ml 70% ethanol twice prior to being dissolved in H_2_O. Additionally, a DNAse digest was performed using DNase I (New England Biolabs, Frankfurt am Main, Germany) in the presence of an RNase inhibitor (RNasin, Promega, Mannheim, Germany) at 37° C for 1 h for removal of residual DNA in the samples. Finally, samples were incubated with 1 µl EDTA (25 mM Stock) at 65° C for 10 min for inactivation of DNAse activity.

Reverse transcription was performed using 1 µg RNA per sample and FIREScript RT cDNA synthesis kit (Solis BioDyne, Tartu, Estonia) according to manufacturer’s instructions. For performance of qPCR HOT Fire Pol Eva Green qPCR Mix Plus (Solis BioDyne, Tartu, Estonia) was used according to standard protocols. Two different primer sets were selected for analysis of *alpl* expression, and *eef1a1l1* as well as *gapdh* were used for normalization. The PCR reactions were performed in technical triplicates with the following protocol: The cDNA was diluted 1:2 with H_2_O after synthesis and 0.25 μl were used per sample. 6.25 μl H_2_O, 2 μl of 5x HOT FirePolR EvaGreenR qPCR Mix Plus and 1 μl of the fwd and rev primer mix (2.5 pmol/μl each) were added. The qPCR reactions were performed using 364-well- plates and the Quantstudio Real-Time PCR system (Life Technologies, Darmstadt, Germany). The following program was used: 95°C 15 min, 40x [95°C, 15 s, 60°C 20 s, 72°C 20 s] followed by the generation of a melting curve using temperatures from 60°C until 95°C. The specific primer sequences are listed in table S1.

**Morpholino *alpl* knockdown**

One-cell stage *nac/tra* zebrafish embryos were injected with solutions comprising of Morpholinos (0.1-0.5 mM each; Gene Tools, LLC, USA), Phenol red (pH 7.0; 0.05% final concentration; for visualization of injection solution) and Fluorescein-Isothiocyanate-Dextran (FITC; Sigma-Aldrich; 1 mg/μl; for visualization of successful and uniform injection in 24 hpf embryos). Positively injected embryos were identified 24 hpf by transient green FITC fluorescence and were further analyzed. We used *nac/tra* embryos for Morpholino injection experiments due to their lack of pigmentation, which is leading to a better visibility of effects. Knockdown experiments were performed either by microinjection of an *alpl* ATG Morpholino (5´-AGCATCCACATTCCCACATCACTGG-3´; GRCz11, chr11:27969125-27969149) or *alpl* Splice Morpholino (5´-GCATGCAGGATGTACCTTAGAGA-3´; GRCz11, chr11:27989387-27989409) of different concentrations into one-cell-stage embryos according to previously published guidelines ^1,2^. In general, injection volumes are adjusted to 1/10 volume of the first cell and were controlled by measurements of droplet size in mineral oil on a micrometer scale slide followed by volume calculation (*V* = 1/6*πd*^3^; mean volume: 8.58 nl) ^3^. According to their molecular weight the following amounts [ng] were injected per embryo:

**Table S2:** Amount of injected Morpholino per embryo.

| Morpholino concentration | 0.1mM | 0.25mM | 0.5mM | molecular weight [g/mol] |
| --- | --- | --- | --- | --- |
| *alpl* Splice Morpholino [ng] | 6.745 | 16.864 | 33.728 | 7862.58 |
| ATG Morpholino [ng] | 7.150 | 17.874 | 35.748 | 8333.01 |
| control Morpholino [ng] | 7.145 | 17.864 | 35.727 | 8328.00 |

The Splice Morpholino was targeted against the donor splice site of *alpl* exon 4. Analysis of Morpholino function was performed by RT-PCR via detection of larger, incorrectly spliced mRNA products or increased detection of intron specific fragments (exon 3 to 5 spanning primers: 413 bp for correctly spliced product, 4922 bp for incorrectly spliced product with stabilized intron; 295 bp for an exon skipped mRNA; exon 4 to intron 4-5 primers: 416 bp product indicates presence of intronic sequence at the cDNA level; cDNA control *eef1a1l1* primers: 220 bp). Consequences of altered *alpl* splicing were investigated via RT-PCR subcloning and subsequent sequencing of single clones. For control injections, a standard control Morpholino was used, targeting the human beta-globulin mRNA (5´-CCTCTTACCTCAGTTACAATTTATA-3´), at a concentration corresponding to the highest used *alpl* Morpholino concentration in the corresponding experiment.

**Determination of AP-activity in protein lysates**

For determination of protein content, 2 µl of protein lysates were pipetted into a transparent 96-well plate and 200 µl of the 1:4 diluted RotiQuant solution was added and incubated at RT for 5 min. A BSA standard (0-8.0 µg) was used for determination of the exact protein content of the samples. All samples were pipetted in technical duplicates. The results were determined using the microplate reader GloMax (Promega, Mannheim, Germany) at 620/450 nm.

**Synthesis of *alpl*-specific riboprobes and ISH against *alpl* mRNA**

For ISH RNA probe synthesis, a fragment of the mRNA sequence of *alpl* (ENSDARG00000015546.12) was cloned (targeting exon 2 to 6; size of the probe: 434 bp) using cDNA of pooled zebrafish embryos (fwd: AATCCGTAAACGCAGGCTGA, rev: GCGTTGTTGTGACGATTCCC), *in vitro* transcribed and labeled with Digoxigenin (DIG RNA Labeling Kit SP6/T7, Merck/Sigma-Aldrich).

Embryos were euthanized by MS-222/Tricaine incubation prior to fixation ^4^. ISH was performed according to previously published protocols ^5^. Embryos (older than 20 hpf) were dechorionized and subsequently fixed in 4% PFA/PBST at 4°C overnight. Embryos were washed 4x 5 min in PBST, gradually transferred to 100% MeOH, and finally stored -20°C. After gradual rehydration from MeOH back to PBST, samples were incubated for 2 x 5 min in PBST on a shaker. Proteinase K digestion was performed as follows (see Tab. S3):

**Table S3:** Conditions of proteinase K digestion

| Developmental stadium | Dilution in PBST s.f. | Incubation time |
| --- | --- | --- |
| 2-8 somites | 10 µg/ml | 2 min |
| 12-16 somites | 10 µg/ml | 3 min |
| 24 hpf | 10 µg/ml | 4 min |
| 48 hpf | 15 µg/ml | 10 min |
| 7 dpf | 10 µg/ml | 25 min |

Afterwards, 2 mg/ml glycine in PBST were used for brief washes in order to stop the enzymatic reaction. Fixation was performed using 4% PFA/PBST for 20 min and then samples were washed in PBST 5 x 5 min each. Pre-hybridization with Hyb-Mix (50% formamid, 5x SSC/water, 0,1% Tween-20, 150 µg/ml Heparin, 5 mg/ml Torula RNA) was performed at 65°C in a water bath for 1 h. Afterwards, samples were incubated with the DIG-labeled *alpl* probes (1:75 in Hyb-Mix, 10 min 80°C prior to use) at 65°C overnight. After removal of the probes, samples were incubated for 2x 30 min with 50% formamide/2x SSCT at 65°C. Then samples were incubated for 30 min in 2x SSCT at 65°C, followed by 2x 30 min in 0.2x SSCT at 65°C and 1 min in PBST at RT. Blocking of non-specific antibody binding was performed with 5% sheep serum in PBST for 1 h at RT, prior to incubation with an anti-DIG AP Fab fragment (Roche/Merck, Darmstadt, Germany) in a dilution of 1:3000 in PBST on a shaker overnight at 4°C. On the following day, the samples were washed with PBST on a shaker 6x 20 min, incubated with staining buffer (0.1 M NaCl, 0.1 M Tris-Cl (pH 9.5), 0.1% Tween-20) 2x 5 min and finally treated with NBT/BCIP staining solution (20 µl NBT/BCIP stock solution (Roche/Merck) in 1 ml staining buffer) until a clear positive signal was visible. The staining reaction was stopped with PBST washes (3x 5 min). Afterwards, samples were transferred step-by-step to MeOH and stored at -20°C.

**ELF 97 staining (AP-activity staining)**

Animals were euthanized using a lethal dose of Tricaine/MS-222. Brains of adult *AB/AB* wildtype animals were extracted and instantly frozen using isopentane (AppliChem GmbH, Darmstadt, Germany) that was cooled in liquid nitrogen. Cryosections (12 µm) were prepared and were attached to superfrost plus slides (Menzel/Thermo Fisher Scientific, Waltham, USA). Freshly prepared cryosections were fixed with 100% acetone for 30 min, washed with PBS/0.2% Tween and 1x with PBS for 5 min. ELF 97 staining was performed by incubation in a 1:40 dilution of ELF 97 phosphatase substrate (Thermo Fisher Scientific, Waltham, USA; product number: E6588) in detection buffer for 30 min and stopped by 2x washing in 25 mM EDTA in PBS. Mowiol (Merck/Sigma-Aldrich, Darmstadt, Germany) without DAPI was used for permanent mounting of sections.

**Combined TNAP ISH and PCNA immunohistochemistry on adult zebrafish brains**

Animals were euthanized using a lethal dose of Tricaine/MS-222. Brains were dissected and fixed in 4% PFA/PBST at 4°C overnight. Afterwards, samples were washed in PBST 4x 5 min prior to a stepwise transfer (25%, 50%, 75%, min. 10 min each) towards 100% MeOH, in which the brain samples were stored at -20°C afterwards. Brains were then rehydrated step-by-step (75%, 50%, 25% MeOH/PBST, 20 min each) prior to 2x 10 min incubation in PBST. Proteinase K incubation was performed in a concentration of 10 µg/ml in PBST for 1 h at RT. The samples were washed with PBST 2x 2 min and post-fixed 20 min with 4% PFA/PBST. Afterwards, four washing steps with PBST (5 min each) were performed prior to 1 h incubation with Hyb-Mix (50% Formamide, 5x SSC, 0.1% Tween20, 150 µg/mL Heparin, 5mg/ml Torula RNA) at 65°C. Then samples were incubated with DIG-labelled probes diluted 1:75 in Hyb-Mix at 65°C overnight (probe treatment prior to use: 10 min 80°C). Now, the protocol was performed analogously to the one that was used for embryos, despite the washing step with PBST on day 3, which was performed 8x 15 min instead of 6x 20 min prior to staining reaction. After completion of the staining reaction, brains were incubated with PBST 3x 5 min and then with 25%, 50%, 75%, 85%, 95% MeOH/PBST, MeOH (2x) for 30 min each at RT~~,~~ transferred step-by-step to 100% MeOH, which was used for storage at -20°C.

Additionally, a PCNA-specific staining was performed on whole brain samples. Briefly, samples were rehydrated step-by-step (75%, 50%, 25% MeOH in PBST, 30 min each) and finally incubated for 5x 15 min in PBST/1% Triton X-100. Afterwards, a blocking step was performed using 10% sheep serum in PBST/1% Triton X-100 for 1 h prior to incubation with the primary antibody for PCNA at 1:200 (P8825, mouse monoclonal, Sigma-Aldrich) dilution in 1% sheep serum/PBST/1% Triton X-100 at 4°C overnight. On the following day, samples were washed 4x 1 h using PBST/1% Triton X-100 prior to incubation with anti-rabbit Alexa 488 (1:1000 in 10% sheep serum in PBST/1% Triton X-100) which was performed at 4°C overnight. On day 3 of the staining procedure, brains were incubated 4x 1 h in PBST/1% Triton X-100. The stained whole brain samples were dehydrated step-by-step (5%, 25%, 50%, 75% EtOH in PBS, 2 min each, to 100% EtOH) and subsequently embedded with the JB-4 embedding kit (Polysciences, Warrington, PA, U.S.) according to manufacturer’s instructions.

**Statistics**

For statistical analysis of the CSPD-assay results a Kruskal-Wallis test with Dunn’s multiple comparison post hoc test was calculated in reference to the respective control w/o Levamisole treatment. ** p < 0.01, * p < 0.05.

For the statistical analyses of possible changes in craniofacial development either a student’s t-test for two independent samples (ANOVA standard distribution was verified) or a Mann-Whitney U-test was used (ANOVA standard distribution could not be verified). The tests were performed using the program OriginPro2017. p/U values are given for comparison between the control group (wt) and incubated embryos at different concentrations. p/U ≤ 0.05 are marked with *, p/U ≤ 0.0001 with ** and p/U ≤ 0.00001 with ***; n = 10 samples per group.

Statistical analysis of qPCR data was calculated using two-sided student t-test, paired; *** p ≤ 0.001, ** p < 0.01, * p < 0.05, n.s.: not significant, ref: reference value.

**Supplementary References**

1 Eisen, J. S. & Smith, J. C. Controlling morpholino experiments: don't stop making antisense. *Development* **135**, 1735-1743, doi:10.1242/dev.001115 (2008).

2 Stainier, D. Y. R. *et al.* Guidelines for morpholino use in zebrafish. *Plos Genetics* **13**, doi:ARTN e1007000 10.1371/journal.pgen.1007000 (2017).

3 Schubert, S., Keddig, N., Hanel, R., Kammann, U. Microinjection into zebrafish embryos (*Danio rerio*) - a useful tool in aquatic toxicity testing? *Environ Sci Eur* **26**, doi:10.1186/s12302-014-0032-3 (2014).

4 Matthews, M. & Varga, Z. M. Anesthesia and euthanasia in zebrafish. *ILAR J* **53**, 192-204, doi:10.1093/ilar.53.2.192 (2012).

5 Thisse, B. & Thisse, C. In situ hybridization on whole-mount zebrafish embryos and young larvae. *Methods Mol Biol* **1211**, 53-67, doi:10.1007/978-1-4939-1459-3_5 (2014).

**Supplementary figures**


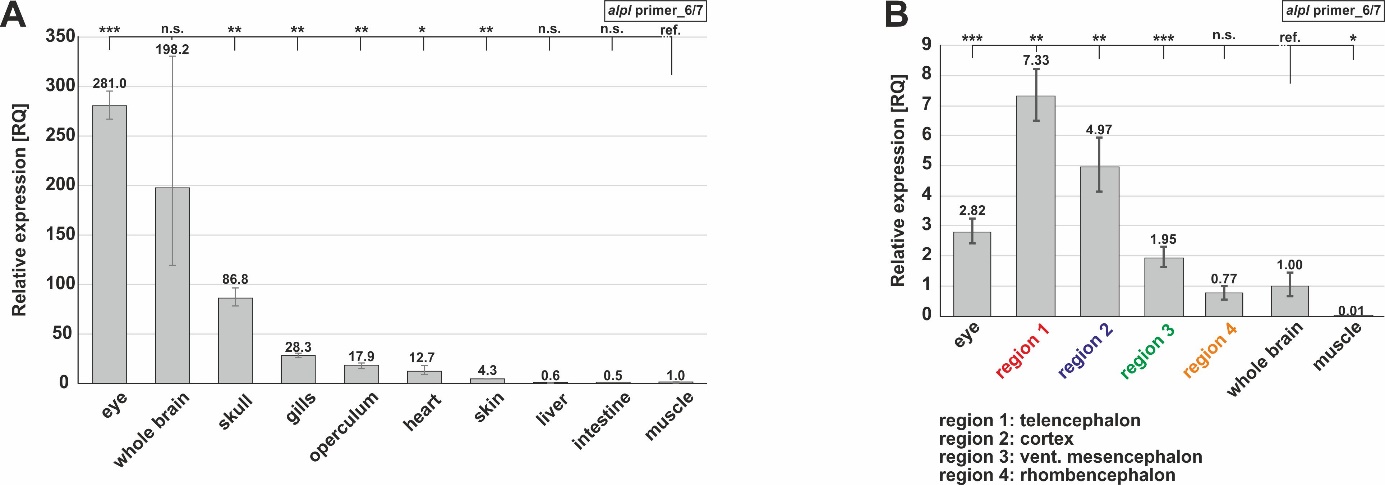


**Fig. S1: Validation of *alpl* expression by qPCR analyses of adult tissues and in different zebrafish brain regions.**

**A:** Relative *alpl* expression in adult tissues. qPCR primers were located in exon 6/7. Normalization was calculated to muscle tissue. n=3 technical replicates. Statistics were calculated using two-sided student t-test, paired; *** p ≤ 0.001, ** p < 0.01, * p < 0.05, **n.s.:** not significant, **ref:** reference. **B:** Relative *alpl* expression in different adult brain regions. qPCR primers were located in exon 6/7 and normalization was calculated using values for whole brain samples. n=3 technical replicates. Statistics were calculated using two-sided student t-test, paired; *** p ≤ 0.001, ** p < 0.01, * p < 0.05, **n.s.:** not significant, **ref:** reference.

**Fig. S2: Control of *alpl* in-situ hybridization specificity.** In-Situ hybridization was performed with zebrafish embryos 24 hpf (A) and 48 hpf (B) incubated with *alpl* anti-sense and corresponding sense probes. Magnifications in the lower row focus on *alpl* expression in the developing eye fields (lateral and dorsal views are shown). Asterisks mark bend tails in 48 hpf embryos, indicating long-time storage artefacts. Scale bars: 100 µm


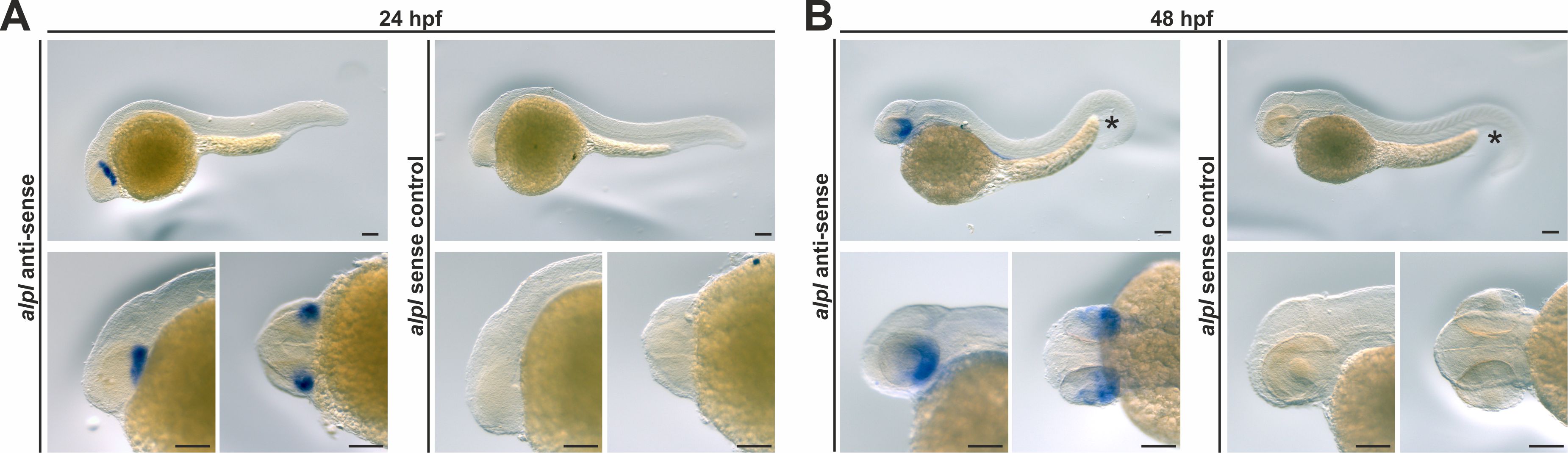


**
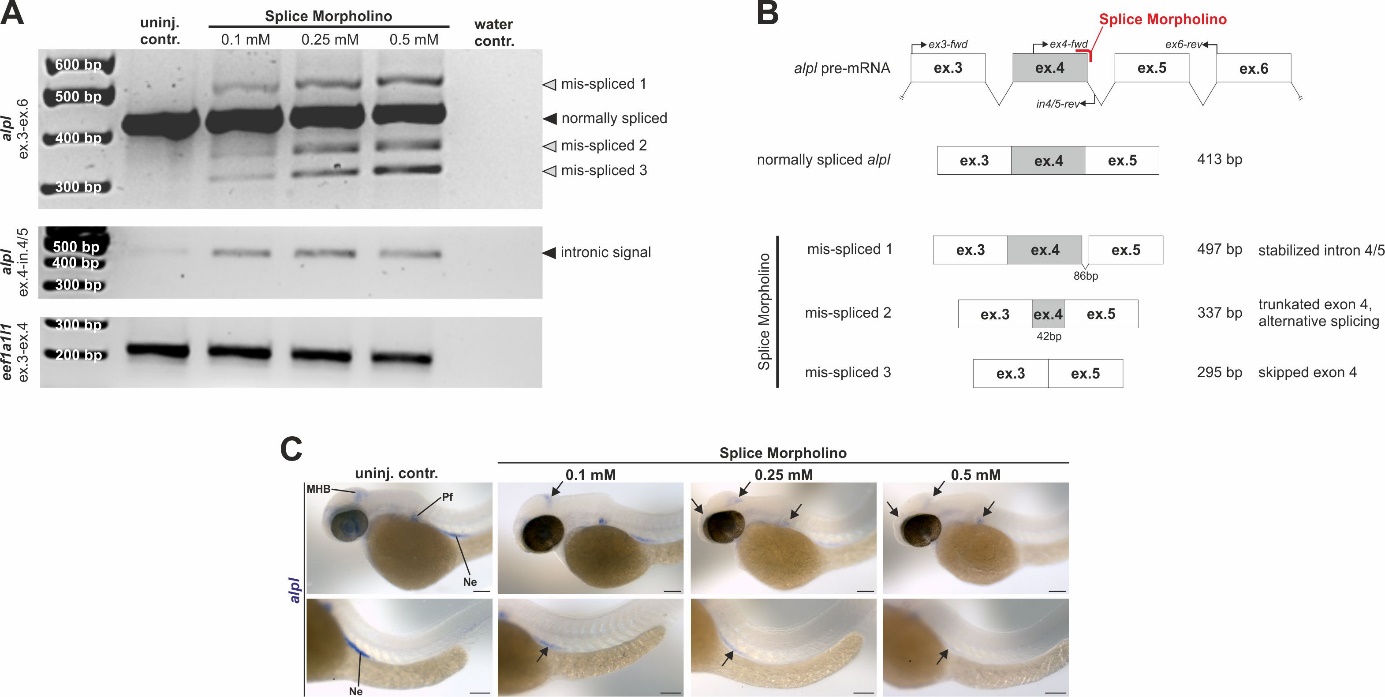
**

**Fig. S3: Validation of Splice Morpholinos against *alpl*.**

**A:** Aberrant splicing products after injection of *alpl* Splice Morpholinos. *eef1a1l1* was used as the control housekeeping gene. Full-length gels are presented in Fig. S16. **B:** Schematic depiction of aberrant splicing processes after injection of *alpl* Splice Morpholinos validated via PCR product sequencing. **C:** ISH for *alpl* in *nac/tra* embryos (48 hpf) after injection of Splice Morpholinos. Black arrows point to positive ISH signals. Scale bars: 100 µm. **Ne:** nephros, **MHB**: mid-hindbrain barrier, **Pf**: pectoral fin.


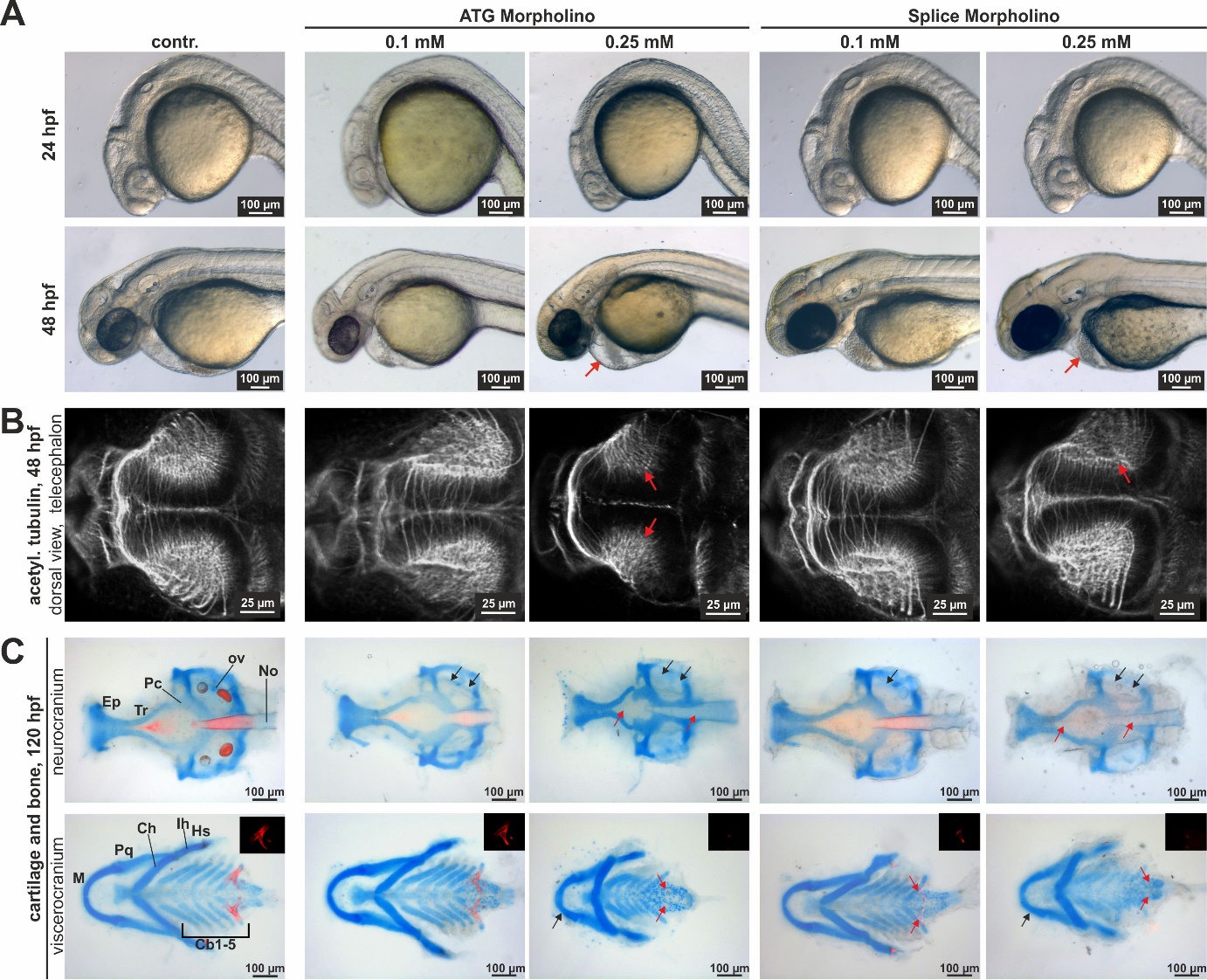


**Fig. S4: Inhibition of *alpl* expression via injection of specific ATG and Splice Morpholinos inhibits axonal development and tissue mineralization. A:** Treatment of zebrafish embryos with different concentrations of Splice and ATG Morpholinos for *alpl* changed normal development at 24 and 48 hpf depending on the respective concentrations. Red arrows indicate enlarged pericardial sac. **B:** Investigation of axonal growth by acetylated tubulin staining detected reduced growth within the optic tectum at 48 hpf. Scale bars: 25 µm. Red arrows point to differences in neural development under Morpholino treatment. **C:** Inhibition of *alpl* expression also resulted in hampered mineralization and influenced craniofacial development at 120 hpf. Alcian-blue stained the cartilage and Alizarin-red stained mineralized structures. Fluorescence signals of Alizarin-red were additionally depicted in the upper right corner. Scale bars: 100 µm. **Cb:** ceratobranchial cartilage, **Ch:** ceratohylal cartilage, **Ep:** ethmoid plate, **Hs:** hysosympletic, **Ih:** interhylal, **M:** Meckel’s cartilage, **No:** notochord, **Ov:** otic vesicle, **Pc:** parachordal cartilage, **Pq:** palatoquadrate cartilage, **Tr:** trabecular cartilage.


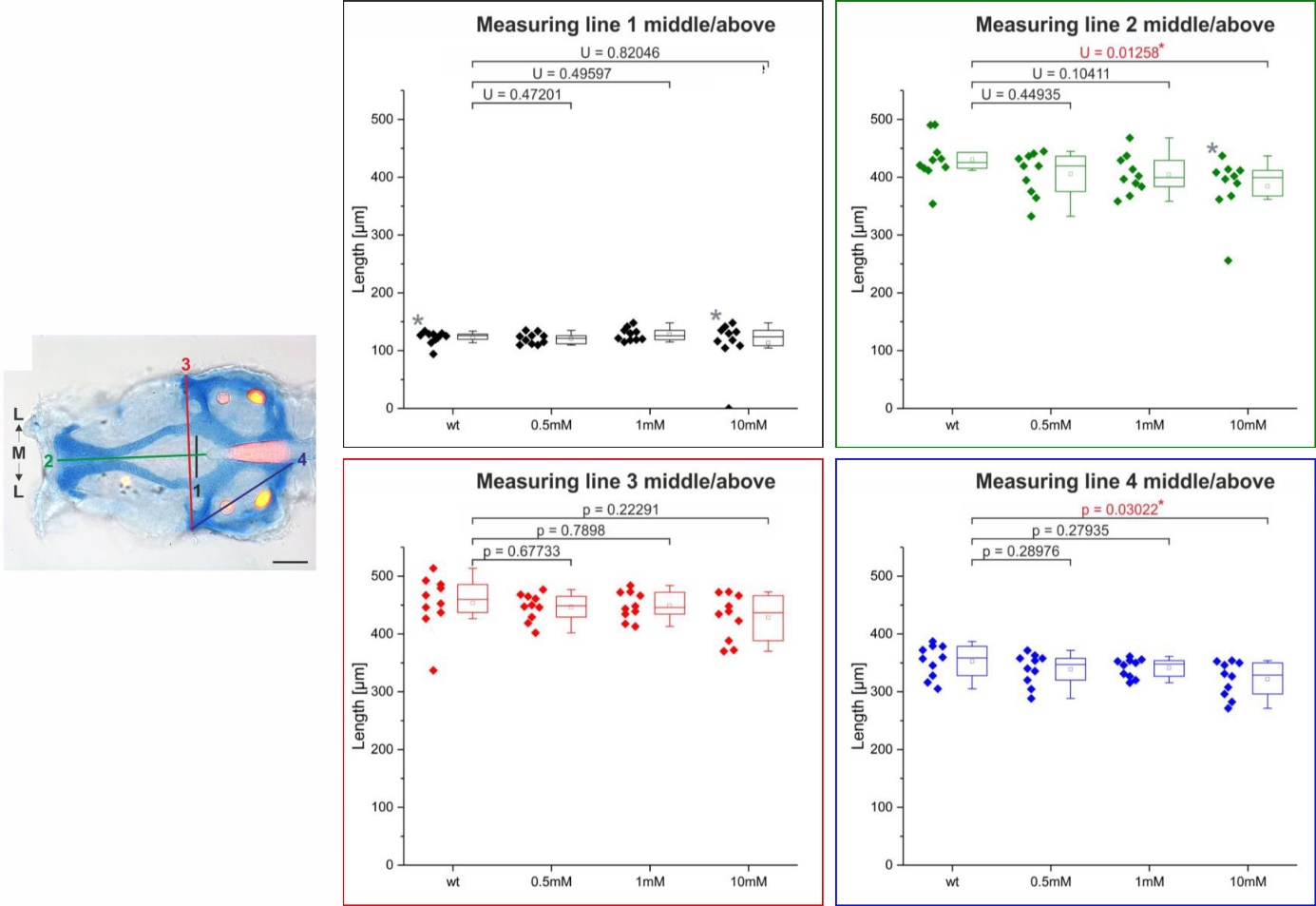


**Fig. S5: Statistical analysis of the cartilaginous neurocranium of Levamisole-incubated embryos at 120 hpf.**

Distance measurements within structures of the neurocranium were performed in Levamisole-incubated embryos at 120 hpf after cartilage and mineralized tissue staining. p/U ≤ 0.05 were marked with *, p/U ≤ 0.0001 with ** and p/U ≤ 0.00001 with ***; n = 10 samples per group.


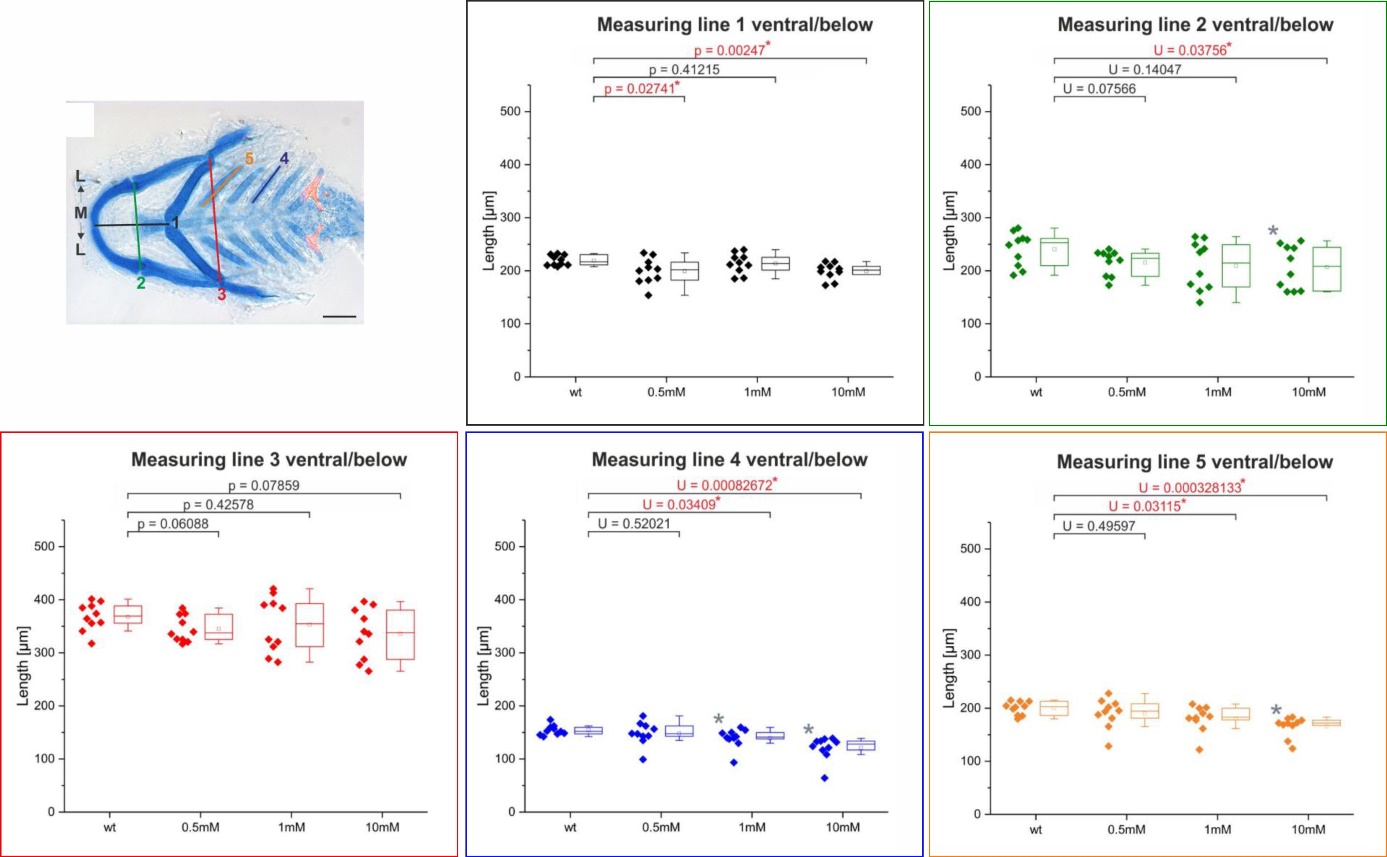


**Fig. S6: Statistical analysis of the cartilaginous viscerocranium of Levamisole-incubated embryos at 120 hpf.**

Distance measurements within structures of the viscerocranium were performed in Levamisole-incubated embryos at 120 hpf after cartilage and mineralized tissue staining. p/U ≤ 0.05 were marked with *, p/U ≤ 0.0001 with ** and p/U ≤ 0.00001 with ***; n = 10 samples per group.


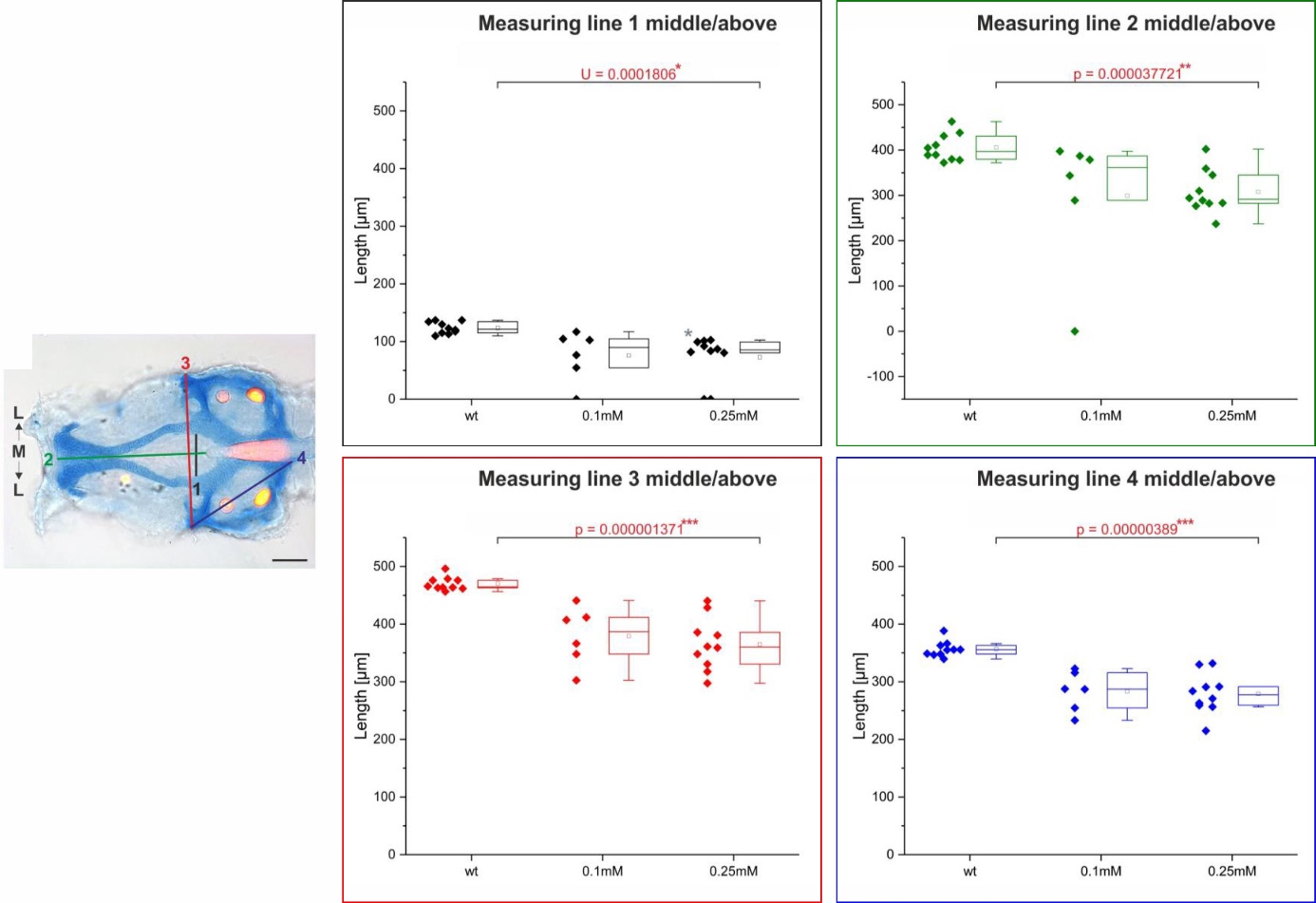


**Fig. S7: Statistical analysis of the cartilaginous neurocranium of ATG Morpholino-treated embryos at 120 hpf.**

Distance measurements within structures of the neurocranium were performed in ATG Morpholino-treated embryos at 120 hpf after cartilage and mineralized tissue staining. p/U ≤ 0.05 were marked with *, p/U ≤ 0.0001 with ** and p/U ≤ 0.00001 with ***; n = 10 samples per group.


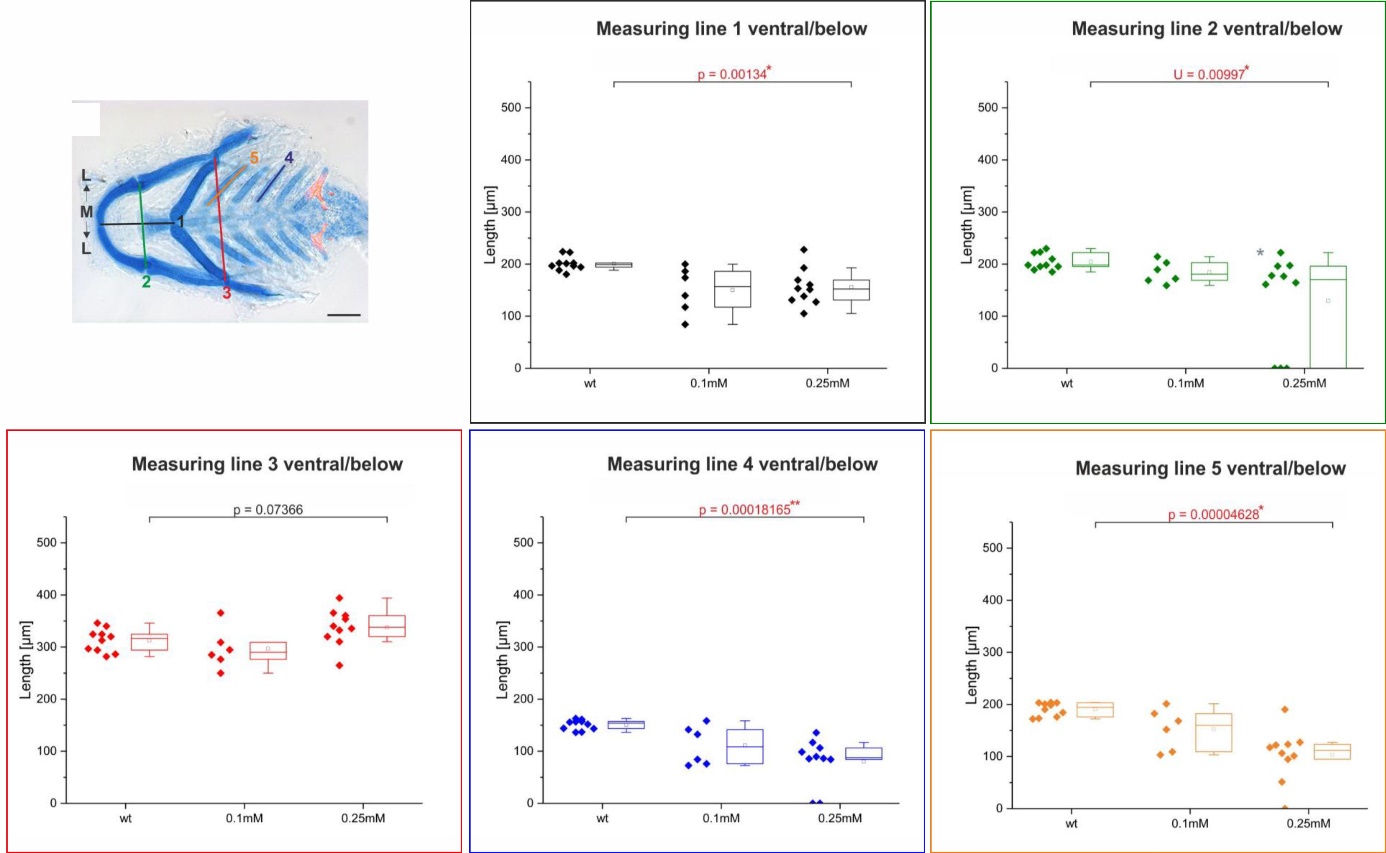


**Fig. S8: Statistical analysis of the cartilaginous viscerocranium of ATG Morpholino-treated embryos at 120 hpf.**

Distance measurements within structures of the viscerocranium were performed in ATG Morpholino-treated embryos at 120 hpf after cartilage and mineralized tissue staining. p/U ≤ 0.05 were marked with *, p/U ≤ 0.0001 with ** and p/U ≤ 0.00001 with ***; n = 10 samples per group (0.1 mM: only 6 samples)


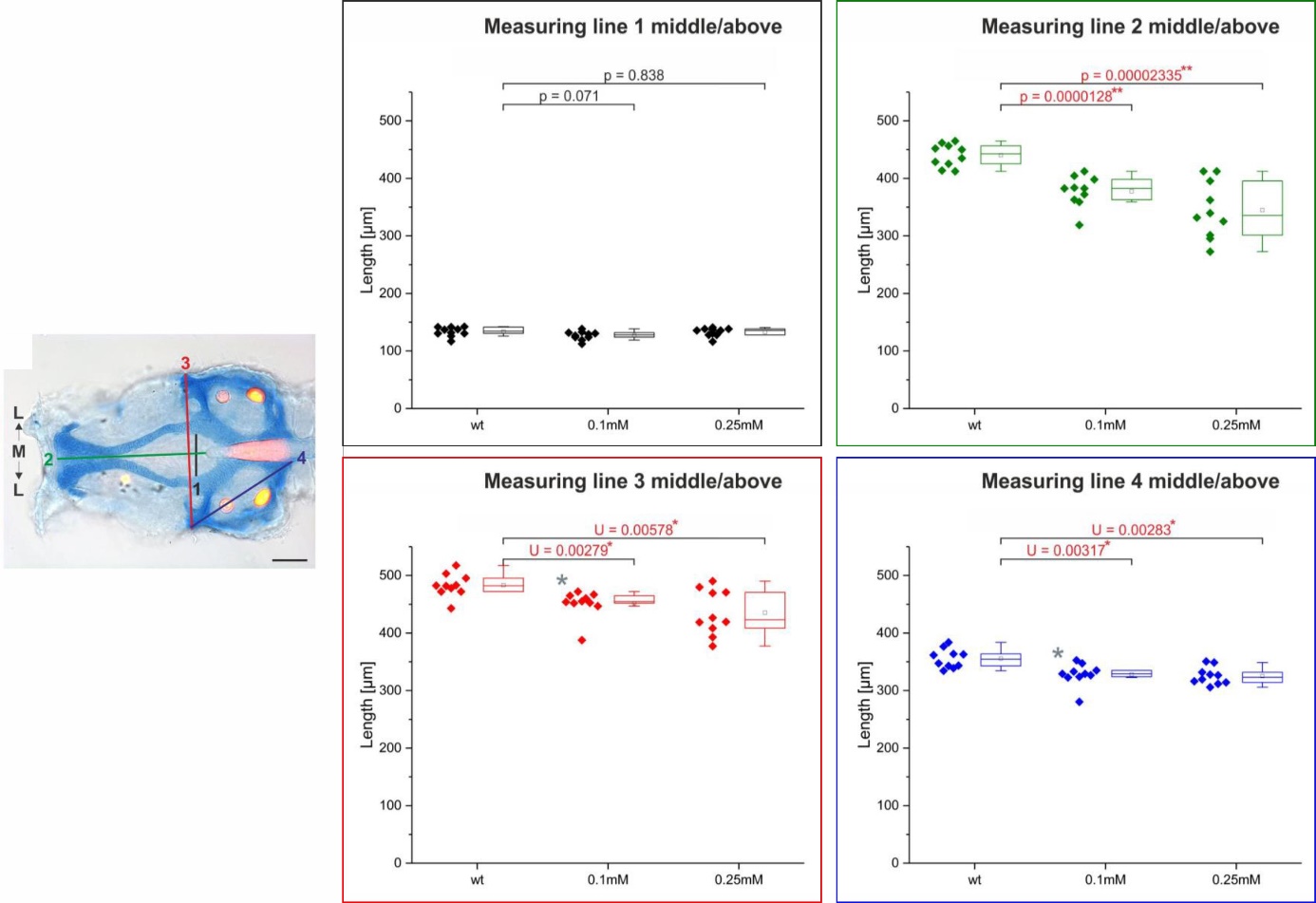


**Fig. S9: Statistical analysis of the cartilaginous neurocranium of Splice Morpholino-treated embryos at 120 hpf.**

Distance measurements within structures of the neurocranium were performed in Splice Morpholino-treated embryos at 120 hpf after cartilage and mineralized tissue staining. p/U ≤ 0.05 were marked with *, p/U ≤ 0.0001 with ** and p/U ≤ 0.00001 with ***; n = 10 samples per group


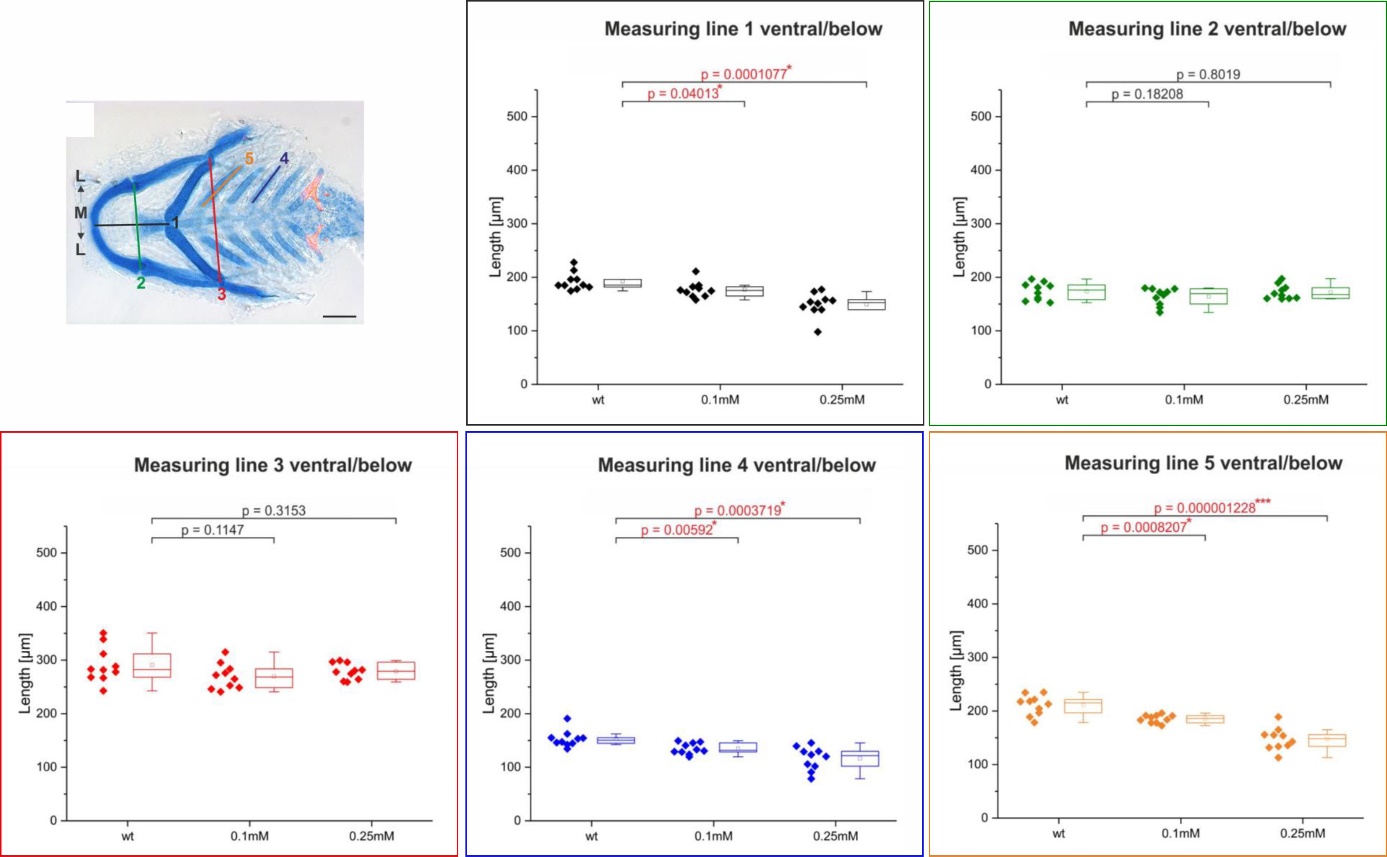


**Fig. S10: Statistical analysis of the cartilaginous viscerocranium of Splice Morpholino-treated embryos at 120 hpf.**

Distance measurements within structures of the viscerocranium were performed in Splice Morpholino-treated embryos at 120 hpf after cartilage and mineralized tissue staining. p/U ≤ 0.05 were marked with *, p/U ≤ 0.0001 with ** and p/U ≤ 0.00001 with ***; n = 10 samples per group


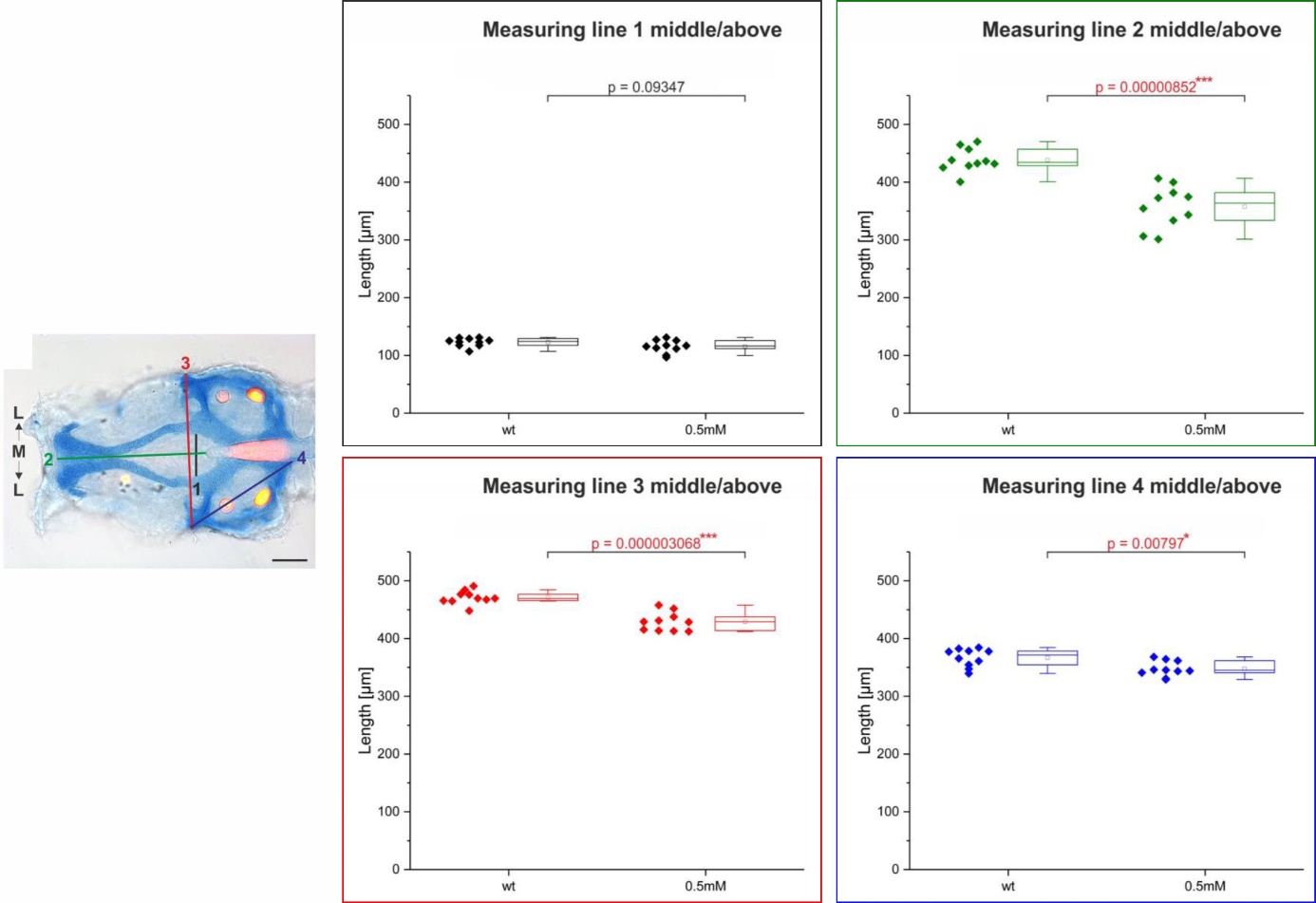


**Fig. S11: Statistical analysis of the cartilaginous neurocranium of control Morpholino-treated embryos at 120 hpf.**

Distance measurements within structures of the neurocranium were performed in control Morpholino-injected embryos (concentration: 0.5mM) at 120 hpf after cartilage and mineralized tissue staining. p/U ≤ 0.05 were marked with *, p/U ≤ 0.0001 with ** and p/U ≤ 0.00001 with ***; n = 10 samples per group


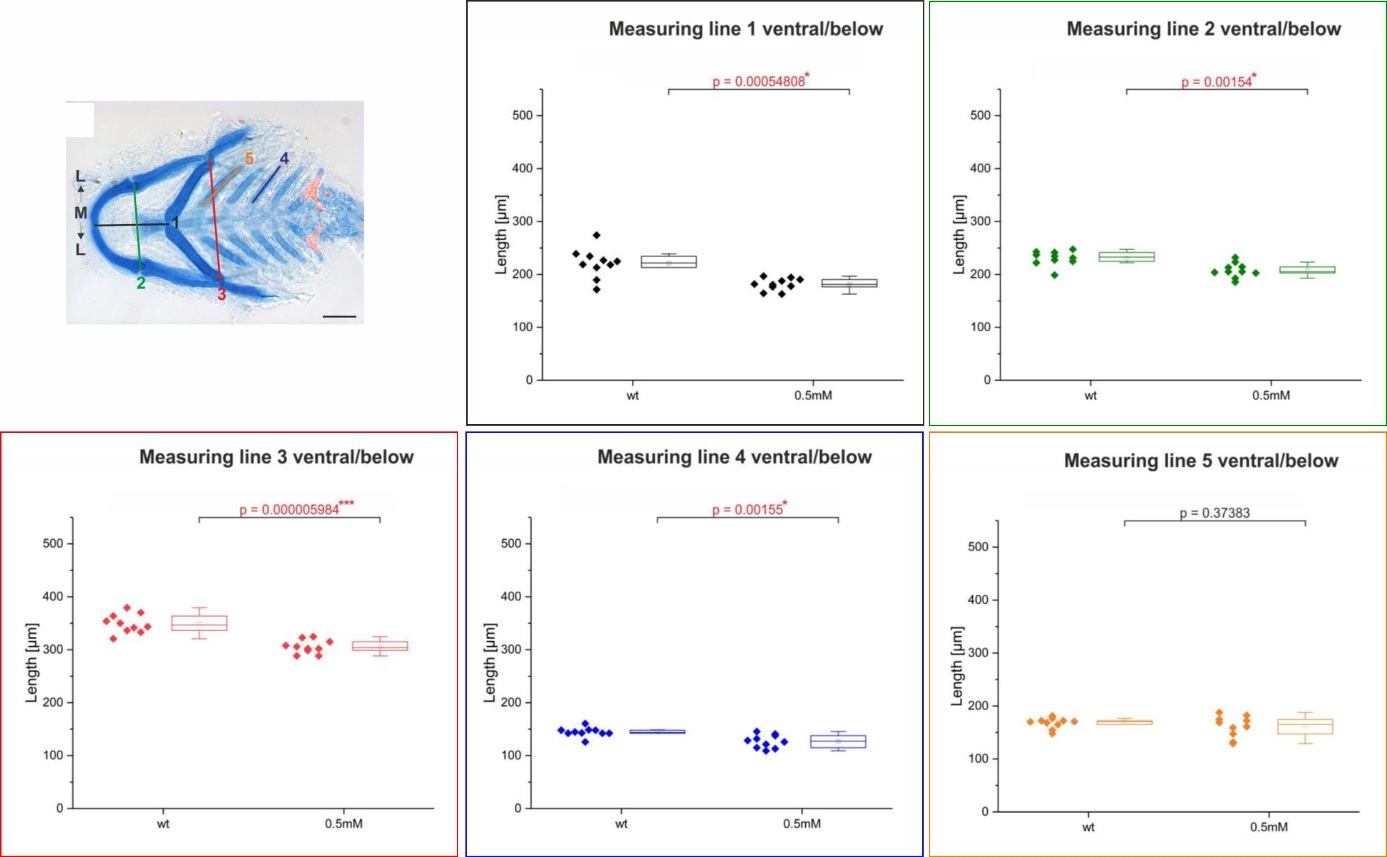


**Fig. S12: Statistical analysis of the cartilaginous viscerocranium of control Morpholino-treated embryos 120 hpf.**

Distance measurements within structures of the viscerocranium were performed in control Morpholino-injected embryos (concentration: 0.5mM) 120 hpf after cartilage and bone staining. p/U ≤ 0.05 were marked with *, p/U ≤ 0.0001 with ** and p/U ≤ 0.00001 with ***; n = 10 samples per group

**
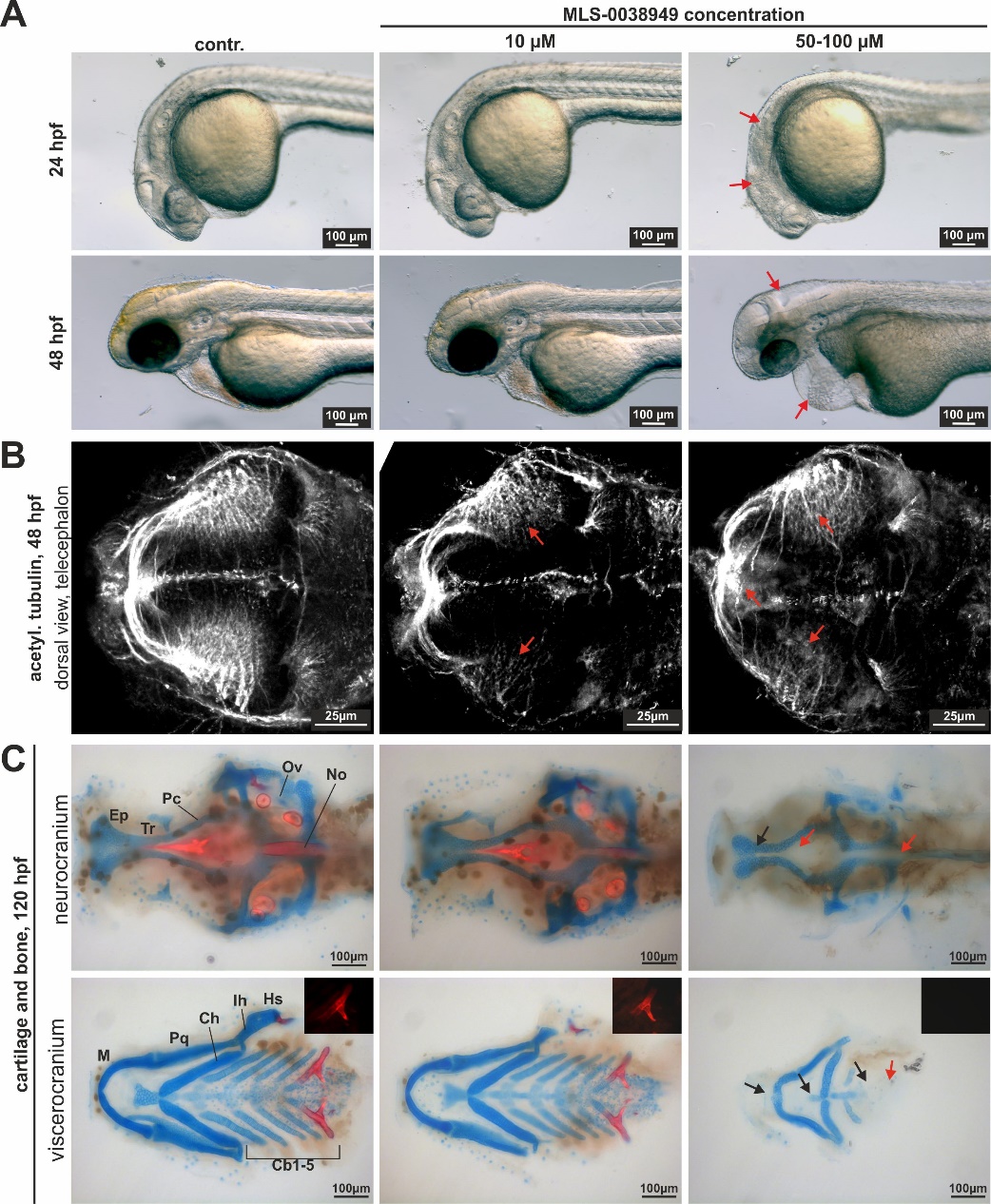
**

**Fig. S13: Inhibition of Tnap-activity via MLS-0038949 incubation inhibits axonal growth and tissue mineralization.**

**A:** Incubation of zebrafish embryos in different concentrations of MLS-0038949 slightly changed the normal development at 24 and 48 hpf depending on its concentration. Red arrows point to differences in development under MLS-0038949 treatment. **B:** Investigation of axonal growth by acetylated tubulin staining implied reduced growth within the optic tectum at 48 hpf. Red arrows point to differences in neural development under MLS-0038949 treatment. **C:** Inhibition of Tnap-activity resulted in hampered mineralization and influenced craniofacial development at 120 hpf. Alcian blue stained the cartilage and Alizarin red stained mineralized structures. Fluorescence signals of Alizarin red were additionally depicted in the upper right corner. Black arrows were used to point out morphological changes of the cartilage and red arrows to mark missing mineralized structures; **Ep:** ethmoid plate, **Tr:** trabecular cartilage, **Pc:** parachordal cartilage, **Ov:** otic vesicle, **No:** notochord, **M:** Meckel’s cartilage, **Pq:** palatoquadrate cartilage, **Ch:** ceratohylal cartilage, **Ih:** interhylal, **Hs:** hysosympletic, **Cb:** ceratobranchial cartilage.


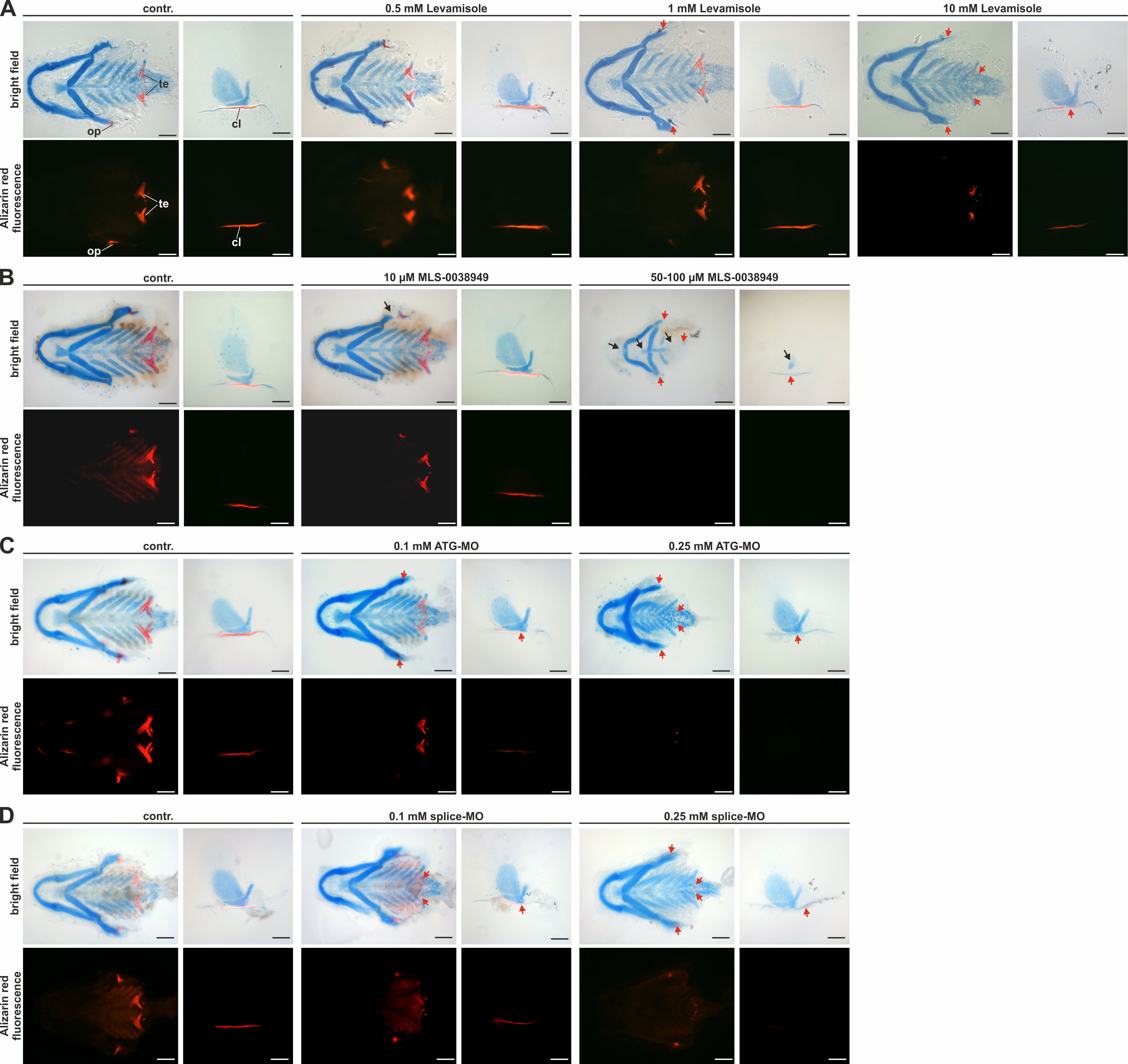


**Fig. S14: Influence of Tnap inhibition or *alpl* knockdown on tissue mineralization.**

Preparations of viscerocranium and pectoral fins of 5 dpf embryos stained for cartilage and mineralized tissue after (A) Levamisole incubation, (B) MLS-0038949 incubation, (C) *alpl* ATG Morpholino knockdown, and (D) *alpl* splice Morpholino knockdown. Reduction of Tnap function and Morpholino knockdown result in reduction or loss of cartilage (marked with black arrows) and mineralized structures (marked with red arrows). Scale bars: 100 µm. **te:** teeth, **op:** operculum, **cl:** cleithrum.


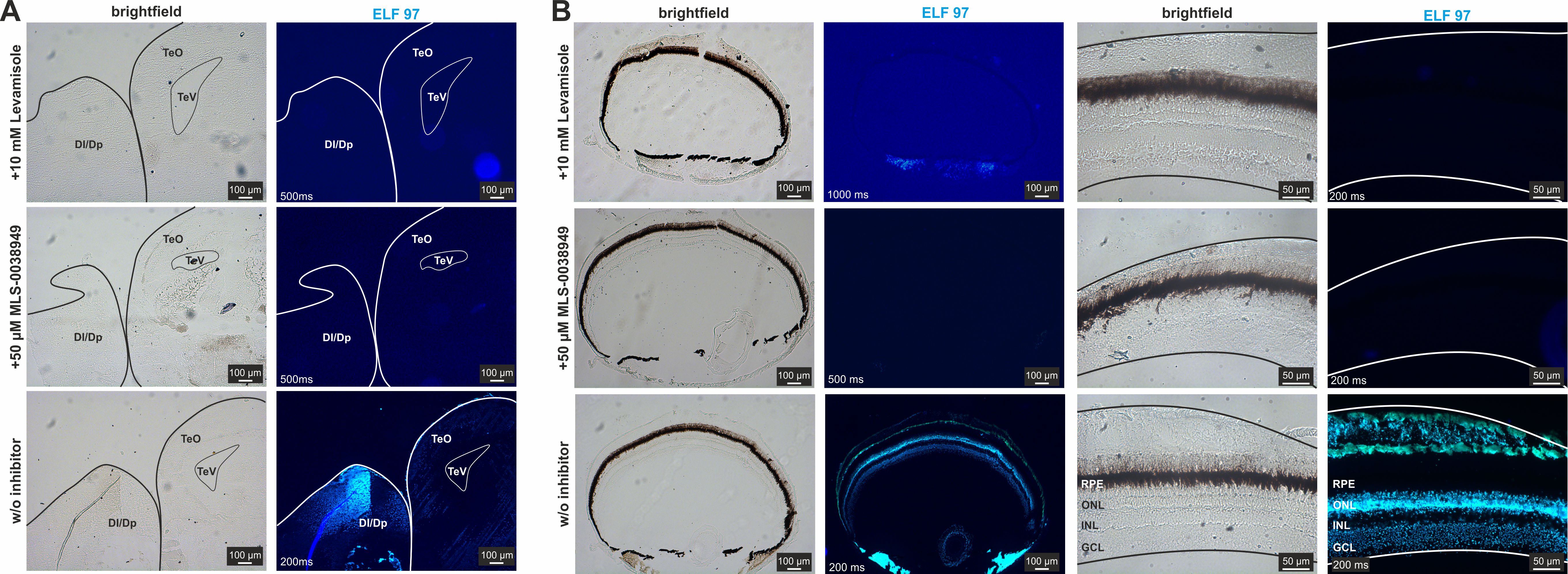


**Fig. S15: Control of ELF 97 staining specificity.**

Whole brain and eye cryosections of adult zebrafish were analyzed for AP-activity via ELF 97 staining in presence of 10 mM Levamisole and 50 µM MLS-0038949. Normal staining was carried out without (w/o) TNAP inhibitor. Application of both TNAP inhibitors resulted in prominent loss of ELF 97 signals in the diencephalon (**A**) and in eyes (**B**). **DI/Dp:** diencephalon lateral zone/posterior zone, **TeO:** tectum opticum, **TeV:** tectal ventricle, **RPE:** retinal pigmented epithelium, **ONL:** outer nuclear layer, **INL:** inner nuclear layer, **GCL:** ganglion cell layer. Given times [ms] indicate illumination times used for imaging ELF 97 signals.


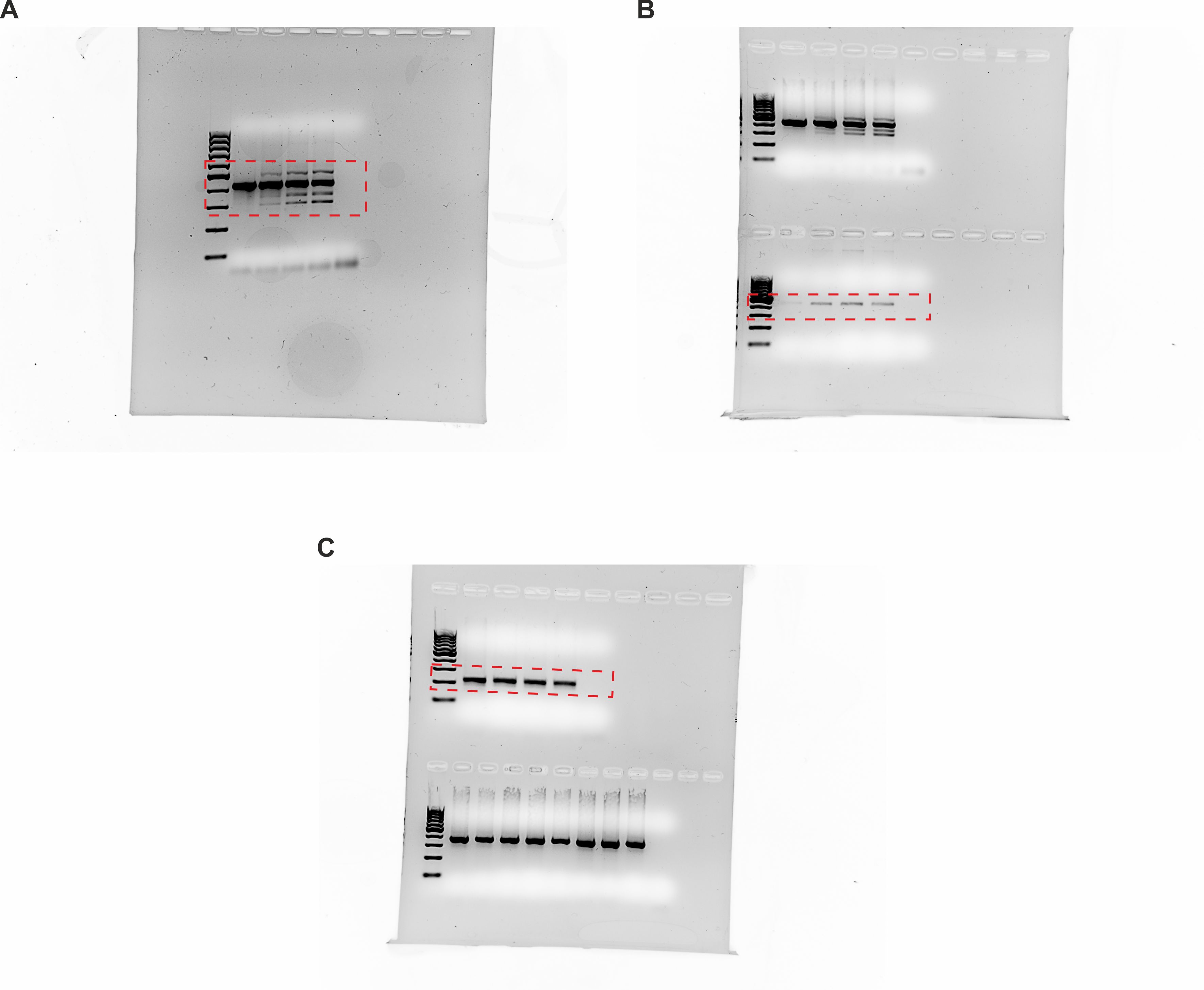


**Fig. S16: Images of full-length gels presented in this manuscript.**

(**A-C**) show unprocessed images of full-length electrophoretic PCR gels partly presented in Fig. S3A (Detection of aberrant splicing products after injection of *alpl* Splice Morpholinos). Dashed red lines indicate cropped areas shown in Fig. S3A.
